# Characterization of the Autophagy Marker Protein Atg8 Reveals Atypical Features of Autophagy in *Plasmodium falciparum*

**DOI:** 10.1371/journal.pone.0113220

**Published:** 2014-11-26

**Authors:** Rahul Navale, Atul, Aparna Devi Allanki, Puran Singh Sijwali

**Affiliations:** Council of Scientific & Industrial Research (CSIR)-Centre for Cellular and Molecular Biology, Hyderabad, AP, India; University of Bern, Switzerland

## Abstract

Conventional autophagy is a lysosome-dependent degradation process that has crucial homeostatic and regulatory functions in eukaryotic organisms. As malaria parasites must dispose a number of self and host cellular contents, we investigated if autophagy in malaria parasites is similar to the conventional autophagy. Genome wide analysis revealed a partial autophagy repertoire in *Plasmodium*, as homologs for only 15 of the 33 yeast autophagy proteins could be identified, including the autophagy marker Atg8. To gain insights into autophagy in malaria parasites, we investigated *Plasmodium falciparum* Atg8 (PfAtg8) employing techniques and conditions that are routinely used to study autophagy. Atg8 was similarly expressed and showed punctate localization throughout the parasite in both asexual and sexual stages; it was exclusively found in the pellet fraction as an integral membrane protein, which is in contrast to the yeast or mammalian Atg8 that is distributed among cytosolic and membrane fractions, and suggests for a constitutive autophagy. Starvation, the best known autophagy inducer, decreased PfAtg8 level by almost 3-fold compared to the normally growing parasites. Neither the Atg8-associated puncta nor the Atg8 expression level was significantly altered by treatment of parasites with routinely used autophagy inhibitors (cysteine (E64) and aspartic (pepstatin) protease inhibitors, the kinase inhibitor 3-methyladenine, and the lysosomotropic agent chloroquine), indicating an atypical feature of autophagy. Furthermore, prolonged inhibition of the major food vacuole protease activity by E64 and pepstatin did not cause accumulation of the Atg8-associated puncta in the food vacuole, suggesting that autophagy is primarily not meant for degradative function in malaria parasites. Atg8 showed partial colocalization with the apicoplast; doxycycline treatment, which disrupts apicoplast, did not affect Atg8 localization, suggesting a role, but not exclusive, in apicoplast biogenesis. Collectively, our results reveal several atypical features of autophagy in malaria parasites, which may be largely associated with non-degradative processes.

## Introduction

Eukaryotic organisms principally rely on two degradation machineries for turnover of dispensable and damaged cellular contents: the ubiquitin proteasome system (UPS) and the lysosomal system. Autophagy is a lysosome-dependent process that delivers a variety of cellular contents, including organelles, to the lysosome, primarily for degradation purpose, and this type of process is regarded as conventional autophagy [1], [2]. However, a number of cellular contents are also selectively delivered to the lysosome for degradative or non-degradative purpose, which is broadly considered selective or unconventional autophagy [3]. If the autophagy cargo contains random cellular contents, it is known as macroautophagy or autophagy; it is called microautophagy (such as mitophagy) if the cargo is selective; it is known as cytoplasm to vacuolar targeting (Cvt) if the cargo is selective and delivered to the lysosome for non-degradative purpose [1], [4], [5]. Thus, autophagy has both housekeeping and regulatory functions in eukaryotic organisms [6]. While autophagy has also been shown to have roles in defence against pathogens, several pathogens also exploit the host autophagy machinery for their advantage [7]. Malaria parasites develop through multiple stages in diverse environments, and this multi-stage development is accompanied by acquisition and disposal of several stage-specific cellular contents, including organelles. As autophagy is involved in both degradative and biosynthetic turnover of cellular contents, it is likely to have key roles in malaria parasite development.

Thirty three autophagy (Atg) proteins participate at different stages of autophagy in *Saccharomyces cerevisiae* [8], [9]. These stages are: initiation of autophagy at a site known as the phagophore assembly site (PAS); cargo selection; vesicle nucleation and assembly at PAS to form a double membrane cup-shaped structure called the phagophore; expansion of phagophore into a double membrane vesicle called the autophagosome; fusion of autophagosome with the lysosome and degradation of its cargo inside the lysosome; and efflux of degradation products into the cytosol [9]. Autophagy is maintained at basal level by the serine/threonine kinase TOR (target of rapamycin) that negatively regulates the autophagy inducer Atg1 complex, which is formed by the Ser/Thr kinases Atg1 and Atg13 and the non-kinase protein Atg17 [10], [11], [12], [13]. The Atg1 complex recruits several Atg proteins at the PAS where a class III phosphatidylinositol 3-kinase (PtdIns3K) complex (Vps34-Vps15-Atg14-Atg6) initiates nucleation and assembly of the phagophore [14], [15]. The PtdIns3K complex generates phospatidylinositol 3-phosphate (PtdIns3P) that binds to several Atg proteins (Atg18, Atg20, Atg21, and Atg24), which are then recruited to PAS. Although not clearly understood, Atg9 has been proposed to carry membranes to the PAS from uncharacterized peripheral sites [16], [17], [18]. Two ubiquitin-like conjugation systems, Atg12-Atg5-Atg16 and Atg8-phosphatidylethanolamine (Atg8-PE), are required for elongation and expansion of phagophore into autophagosome [19]. The formation of Atg12-Atg5-Atg16 and Atg8-PE conjugates requires four additional Atg proteins: Atg4, a cysteine protease that cleaves the carboxy-terminus of Atg8 immediately after a conserved Gly residue; Atg7, a ubiquitin activating E1-like enzyme that activates Atg12 and the processed Atg8; Atg3, an E2-like conjugating enzyme that conjugates phosphatidylethanolamine (PE) to the exposed C-terminus Gly of Atg8 forming the Atg8-PE conjugate; and Atg10, an E2-like conjugating enzyme that conjugates the C-terminus Gly of Atg12 to an internal lysine residue of Atg5 to form the Atg12-Atg5 complex. The cytoplasmic contents and organelles are picked up non-selectively (macroautophagy) or selectively (microautophagy) by specific receptors (Atg11 and Atg19 in yeast and p62/sequestosome-1 in mammalian cells) and delivered to the PAS through interactions with Atg8-PE and the Atg1 complex [20]. Upon completion of the autophagosome formation, the outer membrane of autophagosome fuses with the lysosome, delivering the single membrane enclosed cargo, called the autophagic body, inside the lysosome wherein it is lysed by a lipase (Atg15) and its cargo is degraded by proteases and the degradation products are released into the cytosol through an effluxer (Atg22) [21], [22], [23]. During autophagosome-lysosome fusion, Atg4 cleaves Atg8 off the outer membrane of the autophagosome for reuse. Atg8 is commonly used, and has been accepted as a bonafide marker for investigating autophagy [24], [25], [26].

Genome wide surveys of several protozoa parasites, including *P. falciparum*, have indicated the presence of several key components of the autophagy machinery, and a role for autophagy has been shown in development and life cycle progression of *Trypanosoma*, *Entamoeba*, *Leishmania*, and *Toxoplasma* [27], [28], [29], [30], [31], [32], [33], [34], [35], [36]. Malaria parasites undergo profound morphological and functional changes during its development, which involves at least 15 morphologically and functionally distinct stages, and autophagy may have a crucial role in its development. Autophagy has been suggested to have a role in organelle clearance and morphological changes during liver stage development [37]; multiple independent genome wide analyses have predicted the presence of a partial autophagy machinery in malaria parasites, as the parasite seems to lack almost half of the autophagy proteins known to date [29], [38]. A direct evidence for the presence of autophagy in erythrocytic stages of *P. falciparum* and livers stages of *P. berghei* was provided by five independent groups recently [39], [40], [41], [42], [43]. In the first report, Kitamura *et al* analysed the subcellular localization of Atg8 in *P. falciparum* erythrocytic trophozoite and schizont stages, and reported almost exclusive localization of Atg8 with the apicoplast, a chloroplast-like organelle [39]. Eickel *et al* investigated Atg8 in *P. berghei* liver stages during normal growth and drug treatment, and also found strong colocalization between Atg8 and the apicoplast [40]. The third report by Tomlins *et al* followed Atg8 in *P. falciparum* trophozoite and schizont stages, and showed that Atg8 is associated with autophagosome-like vesicles wherein it colocalized with the late endosomal marker RAB7 [41]. PfAtg8 and PfRAB7 labelled vesicles were observed throughout the parasite, including within or close to the food vacuole. The authors also reported partial colocalization of PfAtg8 with the apicoplast, hence suggested that autophagy in malaria parasites plays a role in apicoplast formation and in endosomal transport to the food vacuole. The fourth report by Cervantes *et al* demonstrated the localization of PfAtg8 throughout the parasite cytosol in both asexual and sexual stages, with partial colocalization with the apicoplast, and proposed a role of autophagy in apicoplast biogenesis [42]. Cervantes *et al* also identified a number of Atg8-interacting cellular contents, including ribosomes, and suggested a role for Atg8 in ribophagy and microphagy of the nucleus. The fifth report by Jayabalasingham *et al* investigated Atg8 and its conjugation system in *P. falciparum* and *P. berghei*. Jayabalasingham *et al* showed that transgenic *P. falciparum* parasites express mcherry-Atg8 throughout the parasite development, which was localized to tubular structures. The same group also reported association of PbAtg8 with the apicoplast in *P. berghei* liver stages. To sum up, all the five publications, in line with each other, showed colocalization of Atg8 and apicoplast-targeted GFP, and suggested a role for autophagy in apicoplast formation. However, the localization pattern of Atg8 was variable depending on the study, suggesting that Atg8 function is not exclusive to the apicoplast. As autophagy is primarily a lysosome-dependent degradation process, a role for autophagy in food vacuole-dependent processes in malaria parasites needs to be addressed.

Autophagy is maintained at basal level during normal growth conditions to maintain homeostasis, but it is remarkably increased during nutrient starvation to support the basic growth requirement of the cell. The lysosome-like degradative organelle of malaria parasite is known as the food vacuole, and it is the site of haemoglobin degradation that generates amino acids for parasite growth [44]. A number of food vacuole-resident haemoglobin-degrading proteases, including the papain-like cysteine proteases falcipains and the aspartic proteases plasmepsins, are potential drug targets [44], [45], [46], [47], [48], [49], [50], [51], [52], [53]. Perturbing the lysosome activity by changing its pH with the lysosomotropic agent chloroquine (CQ) or by inhibiting the activity of resident cysteine (E64) and aspartic (pepstatin) cathepsins has been shown to inhibit autophagic degradation in both yeast and mammalian cells [26], [54]. However, it remains to be investigated if nutrient deprivation by inhibition of the food vacuole degradative process or starvation also affects autophagy in the parasite.

In this study, we first analysed the genome sequences of malaria parasites to identify components of the autophagy repertoire, which predicted 15 proteins, including the autophagy marker Atg8. Expression and localization of native Atg8 was investigated in all asexual and sexual erythrocytic stages of *P. falciparum* to systematically study autophagy. To gain insights into roles of autophagy in parasite development, parasites under nutrient starvation or under stresses or treated with commonly used inhibitors of autophagy were evaluated for effects on Atg8 localization and expression level. Our results indicate a constitutive autophagy in all asexual and sexual erythrocytic stage parasites. Interestingly, in contrast to the conventional autophagy that gets upregulated upon starvation, autophagy in *Plasmodium* appears to be atypical, as it was drastically down regulated in starved parasites. Furthermore, in contrast to the conventional autophagy, all known inhibitors of autophagy did not cause any noticeable change in Atg8 expression and localization.

## Results

### A partial autophagy machinery in *Plasmodium*

Thirty three proteins participate at different stages of autophagy in *S. cerevisiae* [8], [9]. Analysis of the *P. falciparum* genome database using the yeast Atg proteins as queries identified single homologs for 11 and multiple homologs for 4 of the 33 proteins (Table 1), including 8 previously predicted proteins [38], [39]. The predicted *Plasmodium* autophagy repertoire includes proteins involved in induction (Atg1 and Atg17), cargo selection (Atg11), nucleation and assembly of phagophore (Vps34, Vps15, Atg18, and Atg23), expansion of phagophore into autophagosome (Atg3, Atg4, Atg5, Atg7, Atg8, and Atg12), processing and retrieval of Atg8 from the autophagosome (Atg4), degradation of the autophagic body membrane (Atg15), and efflux of the end products (Atg22). The transcription and proteomic data of *P. falciparum* Atg proteins on the PlasmoDB indicates their expression in multiple stages (Table 1), suggesting the presence of autophagy in multiple parasite stages.

**Table 1:** The components of autophagy machinery in *P. falciparum*.

aAtg proteins	*S. cerevisiae* homolog	*P. falciparum* homolog	Identity with *S. cerevisiae* homolog	bExpression	Remark
*Atg1*	P53104	PF3D7_1450000	2.4e-22	E	Serine/threonine kinase; induces autophagy
**Atg2**	P53855	Not identified			Interacts with Atg9
Atg3	P40344	PF3D7_0905700.1	3.3e-40	E	E2-like enzyme; conjugates Atg8 to PE.
Atg4	P53867	PF3D7_1417300	5.0e-09	E	Cysteine protease; cleaves residues downstream of the C-terminal glycine of Atg8 and releases Atg8 off the autophagosome
Atg5	Q12380	PF3D7_1430400	3.4e-05	E	Conjugated to Atg12
**Atg6/VPS30**	Q02948	Not identified			Part of Ptdins 3-kinase complex
VPS34	P22543	PF3D7_0515300	8.5e-75	E	Part of Ptdins 3-kinase complex
VPS15	P22219	PF3D7_0823000	2.2e-14	E	Part of Ptdins 3-kinase complex
Atg7	P38862	PF3D7_1126100	4.0e-51	E	Ubiquitin activating enzyme E1- like; activates Atg8 and Atg12
Atg8	P38182	PF3D7_1019900	3.1e-24	EGS	Ubiquitin-like protein, gets processed by Atg4 downstream of C-terminal Gly
**Atg9**	Q12142	Not identified			Integral membrane protein
**Atg10**	Q07879	Not identified			E2-like enzyme; conjugates Atg12 to Atg5
Atg11	Q12527	PF3D7_0216700.1	4.0e-14	E	Cargo recognition
Atg12	P38316	PF3D7_1470000	3.4e-09	E	Ubiquitin-like protein; conjugated to Atg5 via its C-terminal Gly
**Atg13**	Q06628	Not identified			Modulates Atg1 activity
**ATG14**	P38270	Not identified			Part of the PtdIns 5-kinase complex 1
*ATG15*	P25641	PF3D7_1427100		E	Phospholipase; breaks down autophagosome membrane
**Atg 16**	Q03818	Not identified			Component of Atg12-Atg5 complex
*Atg17*	Q06410	PF3D7_1120000	1.4e-10	EG	Modulates Atg1 activity
Atg18	P43601	PF3D7_1012900	5.1e-31	EG	Interacts with Atg2
**Atg19**	P35193	Not identified			
**Atg20**	Q07528	Not identified		E	Selective autophagy
**Atg21**	Q02887	Not identified			Required for selective autophagy
*Atg22*	P25568	PF3D7_0629500	1.3e-44	EG	Vacuole effluxer
ATG23	Q06671	PF3D7_0216700.1	5.8e-07	EG	Required for selective autophagy
**Atg24/SNX4**	P47057	Not identified			Required for selective autophagy

a Multiple homologs were predicted for the Atg proteins in italicized font, and homologs could not be identified for Atg proteins in bold font. Atg proteins predicted in previous analyses are underlined [38], [39].

b The *Plasmodium* genome database indicates the presence of transcript and/or peptides for the corresponding *P. falciparum* proteins in erythrocytic (E), gametocyte (G), and sporozoite (S) stages.

Our searches did not identify single homologs for human or yeast Atg1 and Atg17, which together with Atg13 form the Atg1-Atg13-Atg17 complex. ScAtg1 showed high homology with several *P. falciparum* protein kinases (e-value: 4.3e-05 to 2.4e-22), including 16 Ser/Thr kinases. However, none of the 16 Ser/Thr kinases showed domain organization similar to that of ScAtg1 that contains an amino-terminus Ser/Thr kinase domain and a DUF3543 domain of unknown function in the carboxy-terminus. This domain organization is also shared by Atg1 proteins of human (ULK1and ULK2), Drosophila (DAtg1), and *C. elegans* (CeAtg1). PF3D7_1450000 is the closest homolog of ScAtg1 (2.4e-22), but it is about half the size of ScAtg1 (PF3D7_1450000: 376 amino acids, ScAtg1: 897 amino acids). Thus, functional studies would be required to identify the *P. falciparum* Atg1. ScAtg11, a cargo selection protein, and PfAtg11 are 16.3% identical, and both the proteins have 4 putative coiled-coil regions, which have been shown to mediate interaction of ScAtg11 with several proteins (Figure S1) [20].

Vps34 and Vps15, two of the four proteins of the PtdIns3K complex in yeast (Vps34, Vps15, Atg6, and Atg14), are present in *Plasmodium*. PfVps34 shares phosphatidylinositol 3-phosphate kinase accessory and phosphatidylinositol 3-kinase domains with ScVps34 (Figure S2), and has recently been suggested to be involved in transport of haemoglobin from the erythrocyte cytoplasm to the parasite food vacuole [55]. PfVps15 also contains a putative kinase domain (Figure S3). Several yeast Atg proteins bind to phosphatidylinositol 3-phosphate (PtdIns3P) via a conserved Phe-Arg-Arg-Gly-Thr motif (Atg18 and Atg21) or via a Phox homology (PX) domain (Atg20 and Atg24). PfAtg18 contains a conserved PtdIns3-binding motif (Phe-Arg-Arg-Gly-Thr), a WD40 domain, and an Atg8 family interacting motif (AIM) or the LC-interacting region (LIR) (Figure S4) [56], [57]. AIM-containing proteins interact with Atg8-family proteins via the AIM, which is crucial for their recruitment in the autophagy pathway.

The most complete subset of the autophagy repertoire in *Plasmodium* is the ubiquitin-like conjugation systems, which contains 6 of the 8 proteins (Atg3, Atg4, Atg5, Atg7, Atg8, and Atg12; Atg16 and Atg10 could not be identified). The Atg8 conjugation system is complete, and contains Atg8 and the enzymes for its processing and recycling (Atg4), activation (Atg7), and conjugation to PE (Atg3) (Figures S5, S6, S7, S8). The *Plasmodium* Atg12-Atg5-Atg16 conjugation system seems to lack Atg10 and Atg16. It requires Atg7 for activation of Atg12, Atg10 for conjugation of the C-terminus Gly residue of Atg12 to an internal lysine residue of Atg5 to form Atg12-Atg5 complex, which then binds to Atg16 to form the complete complex. Both hAtg12 and ScAtg12 have Gly at the carboxy-terminus end, which is essential for their conjugation to Atg5, but PfAtg12 and its other *Plasmodium* homologs lack Gly at the end (Figure S9). PfAtg5 seems to be a distant homolog of ScAtg5 (7.4% identity), but it contains a positionally conserved lysine residue that in hAtg5 has been shown to be conjugated to the C-terminus Gly of Atg12 (Figure S10).

Upon reaching inside the vacuole, the autophagic body is lysed by Atg15, its cargo is degraded by proteases, and amino acids are effluxed by Atg22. The *P. falciparum* genome encodes 20 putative lipases (PlasmoDB), but only 9 of these are present in all *Plasmodium* species and the remaining 11 are present in *P. falciparum* only. A conserved lipase (PF3D7_1427100), which shares an N-terminus hydrophobic region and a C-terminus lipase domain with ScAtg15, may function as Atg15. The *P. falciparum* food vacuole contains multiple classes of proteases, which have been shown to degrade haemoglobin [44], and some of these may degrade the autophagy cargo. Several proteins are annotated as transporters on the PlasmoDB, including 5 putative amino acid transporters, but none of these showed significant similarity with ScAtg22. Cellular localization studies are necessary to determine which of these transporters are present in the food vacuole. Autophagy is also adapted for selective transport of proteins and organelles to the lysosome [4], [5], [58]. Atg11 together with several other Atg proteins (Atg9, Atg19, Atg20, Atg21, Atg23, and Atg24) is required for delivery of aminopeptidase-1 to the vacuole via selective autophagy in *S. cerevisiae* [59], [60], [61], [62], [63], [64]. The presence of Atg11 and Atg23 homologs in *P. falciparum* suggests the presence of selective autophagy, which may have a role in transport of certain food vacuole proteins and/or organelles. PfAtg23 contains a putative AIM that suggests a role for it in autophagy (Figure S11).

All *P. falciparum* Atg proteins have homologs in other *Plasmodium* species. A comparison of the sequences of identified *P. falciparum* Atg proteins with homologs in other Plasmodium species showed 29.5–97.5% sequence identity (Table S1).

### PfAtg8 is constitutively expressed in asexual erythrocytic stages

**Figure 1:**
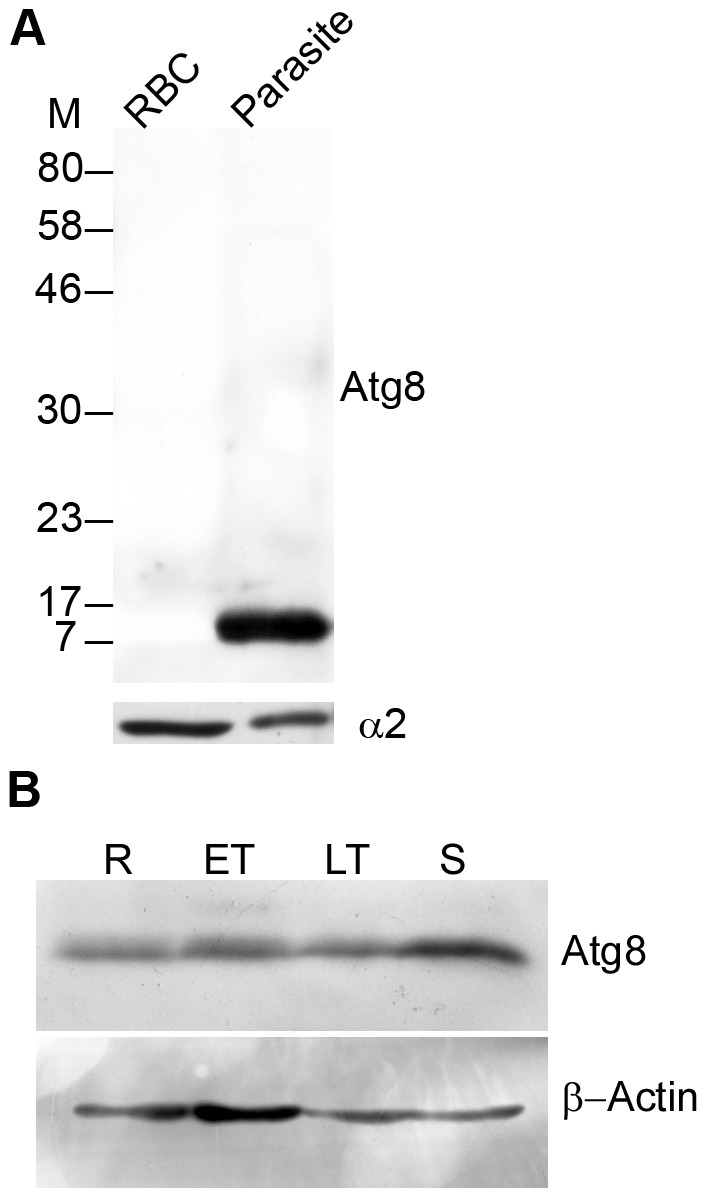
Specificity of anti-PfAtg8 antibody and expression of native PfAtg8. **A**. Western blot of uninfected erythrocyte (RBC, approximately 1×10^8^/lane) and mixed stage parasite (Parasite, approximately 1×10^8^/lane) lysates with anti-Atg8 antibodies showed a single band around the predicted size of native PfAtg8 (∼14 kD) exclusively in the parasite, confirming specificity of the antibodies. Antibodies against the α2 subunit of the proteasome were used as a loading control, which showed similar intensity signal in both the samples. **B**. Western blot of ring (R), early trophozoite (ET), late trophozoite (LT), and schizont (S) stage lysates of *P. falciparum* (approximately 1×10^8^ parasites/lane) with anti-Atg8 (Atg8) and anti-β-actin (β-Actin) antibodies. The blot showed expression of PfAtg8 and the control protein (β-actin) in all stages. M, molecular weight in kD.

Atg8 is routinely used as a marker to study autophagy [24], [25], [26]. To investigate autophagy in *P. falciparum*, recombinant PfAtg8 was produced using an *E. coli* expression system (Figure S12A); antibodies were generated against the recombinant protein, and assessed for specificity for recombinant and native Atg8. Anti-Atg8 antibodies reacted with recombinant Atg8 (Figure S12B), did not react with the uninfected erythrocyte lysate and detected a single band of the expected size of native PfAtg8 (∼14 kDa) in the parasite lysate (Figure 1A), which indicated their specificity for Atg8. The antibodies detected Atg8 in lysates of ring, early and late trophozoites, and schizont stages, indicating the expression of Atg8 in all these stages (Figure 1B); the Atg8 expression level was slightly more in schizonts compared to other stages. Both ScAtg8 and LC3 are processed at the C-terminal by Atg4 that results in a Gly residue at the C-terminus end of the processed Atg8, which is then conjugated to PE. Thus, there are unprocessed Atg8 and the processed Atg8-PE forms of Atg8, and the increase in Atg8-PE levels is considered induction of autophagy. However, we reproducibly observed a single Atg8 band in immunoblots of parasite lysates.

### Atg8 is associated with punctate structures

**Figure 2:**
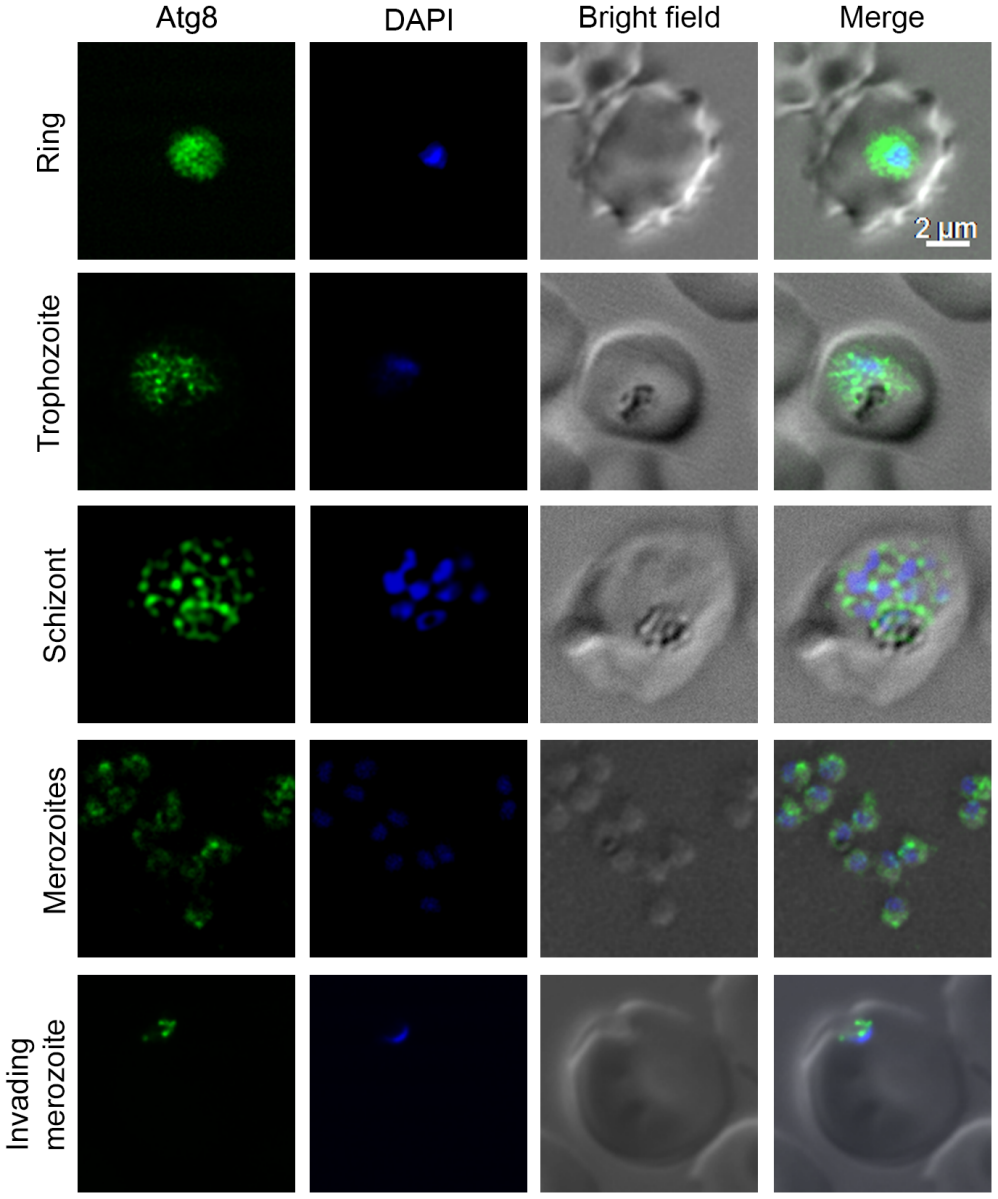
Expression and localization of Atg8 in asexual erythrocytic stages. The indicated stages of *P. falciparum* were evaluated for the presence of Atg8 by IFA using anti-Atg8 antibodies as described in Materials and Methods section. The images of each indicated stage show the presence of Atg8 specific signal (Atg8), nucleic acid staining (DAPI), the parasite and the erythrocyte boundaries (Bright field), and the merged of all three images (Merge). The Atg8 signal is present throughout the parasite in all the stages shown, and appears to be associated with punctate structures, most likely autophagosomes. The scale bar shown is identical for this and all the figures containing IFA images.

To determine localization of PfAtg8, asexual and sexual stages of *P. falciparum* were analysed by immunofluorescence assay (IFA) using anti-Atg8 antibodies. The antibodies showed signal in all asexual and sexual erythrocytic stages (Figures 2 and S13), and the signal was associated with punctate structures, indicating that PfAtg8 is constitutively expressed and is part of vesicles, most likely autophagosomes. It is noteworthy that almost all Atg8 signal was associated with punctate structures throughout the parasite.

**Figure 3:**
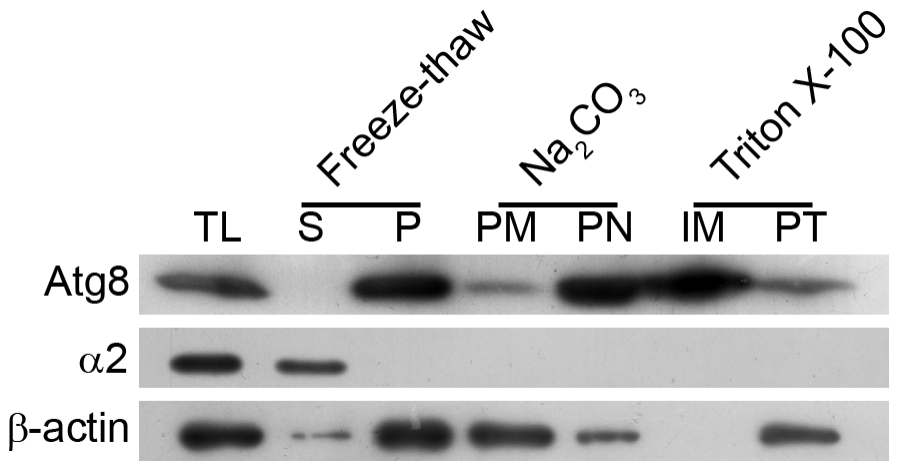
Distribution of Atg8 in different subcellular fractions. *P. falciparum* trophozoite/schizont stage parasites were first lysed by freeze-thaw method, and equal aliquots of the lysate were processed for total freeze-thaw lysate (TL), soluble (S) and pellet (P) fractions of the freeze-thaw lysate, extraction of peripheral membrane proteins (PM) with Na_2_CO_3_, and extraction of integral membrane proteins (IM) with Triton X-100 as described in Materials and Methods section. Equivalent amounts of all the fractions, including the pellets remaining after Na_2_CO_3_ (PN) and Triton X-100 (PT) extractions, were analyzed for the presence of Atg8, the proteasome α2 subunit (α2), and β-actin (β-actin) by western blotting as described in Materials and Methods section. The blot showed predominant Atg8 signal in the total freeze-thaw lysate, the pellet fraction of the freeze-thaw lysate, and the integral membrane protein fraction. Soluble fraction of the freeze-thaw lysate did not have any Atg8 signal and the peripheral membrane fraction had very low signal, indicating that Atg8 is exclusively present in the freeze-thaw pellet fraction, mostly as an integral membrane protein. The α2 was used as a marker for soluble protein, and it is exclusively present in the soluble fraction of the freeze-thaw lysate. β-actin was used as a general control and it appears to be predominantly exist as a peripheral membrane protein. The experiment was repeated three times and the results were reproducible.

Since immunoblotting detected a single band and IFA showed association of almost all the Atg8 signal with punctate structures, we conjectured that Atg8 is membrane-associated. To determine if that is the case, trophozoite/schizont stage parasites were fractionated into soluble, insoluble, peripheral membrane, and triton X-100 extractable fractions, and each fraction was evaluated for the presence of Atg8 and a soluble protein marker (α2 subunit of the proteasome). Almost all of the Atg8 was detected in the insoluble fraction; a small fraction of that was present in the peripheral membrane fraction, and almost half of that was in the triton X-100 extractable integral membrane fraction (Figure 3). The presence in the insoluble fraction of the parasite lysate, a single form in immunoblots, and association with punctate structures together indicate that PfAtg8 is membrane-associated.

### Localization of Atg8 is conserved across *Plasmodium* species

**Figure 4:**
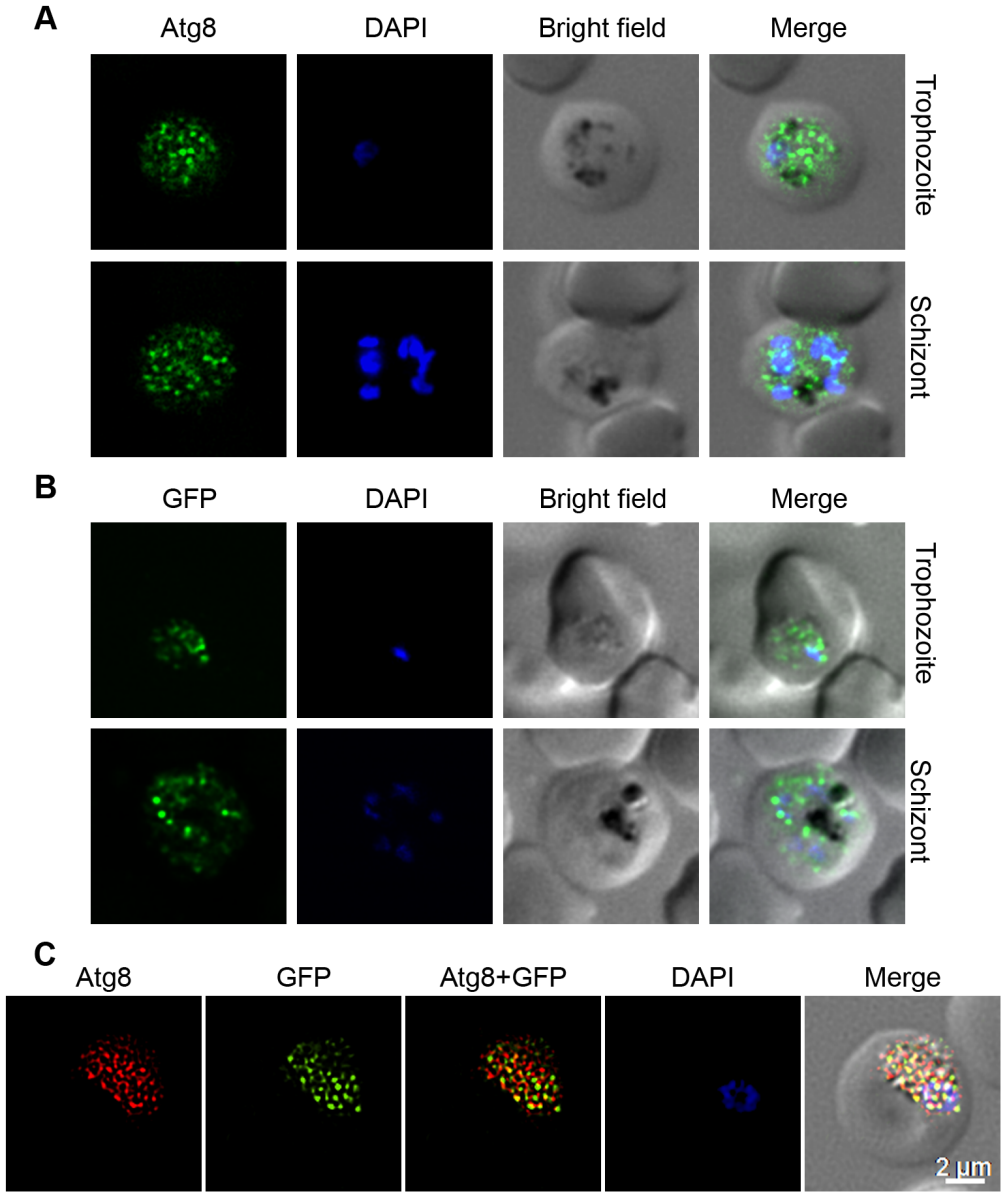
Expression and localization of native and episomally expressed Atg8 in *P. berghei*. The trophozoite and schizont stages of wild type *P. berghei* were evaluated for expression and localization of native PbAtg8 using anti-Atg8 antibodies (A). Similarly, recombinant *P. berghei* parasites were assessed for localization of episomally expressed GFP-PfAtg8 (B) using anti-GFP antibodies and for colocalization of the signals (C) with anti-PfAtg8 and anti-GFP antibodies as described in Materials and Methods section. The images show signal for native PbAtg8 (A; Atg8) or episomally expressed GFP-PfAtg8 (B; GFP) or colocalization of anti-Atg8 and anti-GFP antibody signals (C; Atg8+GFP), staining of the nucleus (DAPI), the parasite and the erythrocyte boundaries (Bright field), and the merged of all three images (Merge). The punctate signal for both native and episomally expressed Atg8 throughout the parasite is similar to the localization pattern of native PfAtg8 in Figure 2. The yellow spots (Atg8+GFP) in C indicate that both the antibodies label the same structures and that anti-PfAtg8 antibodies are specific to PfAtg8. For representation purpose, the GFP signal was false-coloured to green and the Atg8 signal was false-coloured to red.

As all the putative *P. falciparum* Atg proteins have homologs in other malaria parasites (Table S1), we investigated whether the autophagy observed in *P. falciparum* under in vitro conditions is also present under in vivo conditions. We determined localization of native PbAtg8 and episomally-expressed GFP-PfAtg8 in *P. berghei* parasites. As PfAtg8 and PbAtg8 are 87.9% identical, anti-PfAtg8 antibodies were expected to detect PbAtg8. Anti-Atg8 antibodies reacted with native PbAtg8, and showed punctate signal (Figure 4A). The GFP fluorescence of GFP-PfAtg8 was extremely weak in live cells, hence, these parasites were probed with anti-GFP antibodies, which also showed punctate signal (Figure 4B). Furthermore, the signals with anti-PfAtg8 and the anti-GFP antibodies showed strong colocalization, indicating that both the antibodies label the same structures and that anti-PfAtg8 antibodies are specific (Figure 4C). Thus, similar localization patterns of native Atg8 in *P. falciparum* under in vitro culture conditions, in *P. berghei* under in vivo conditions, and that of episomally-expressed GFP-PfAtg8 indicate that autophagy is conserved and operational under both in vitro and in vivo conditions, and all malaria parasites most likely have similar autophagy.

### Atg8 shows partial colocalization with the apicoplast

**Figure 5:**
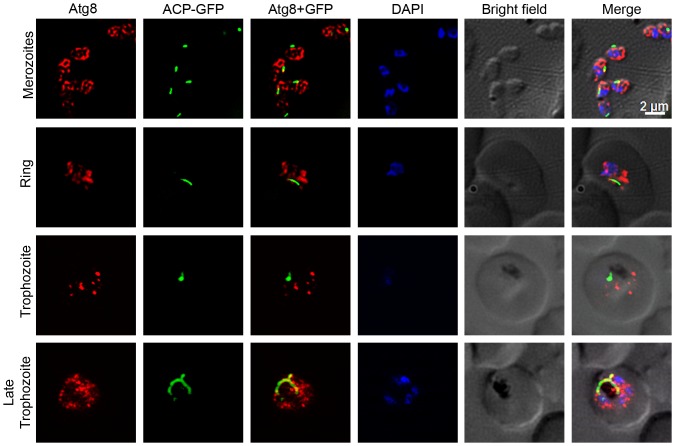
Colocalization of Atg8 and the apicoplast. The indicated stages of *P. falciparum* D10 parasites expressing the apicoplast marker ACP-GFP were evaluated for colocalization of PfAtg8 with the apicoplast as described in Materials and Methods section. The images show PfAtg8 (Atg8) and apicoplast (ACP-GFP), merged Atg8 and apicoplast signals (Atg8+GFP), nuclear stain (DAPI), parasite and erythrocyte boundaries (Bright field), and the merged of all the four images (Merge). Note that PfAtg8 signal is distributed throughout the parasite as puncta, which partially overlaps with the apicoplast, particularly in the late trophozoite stage.

**Figure 6:**
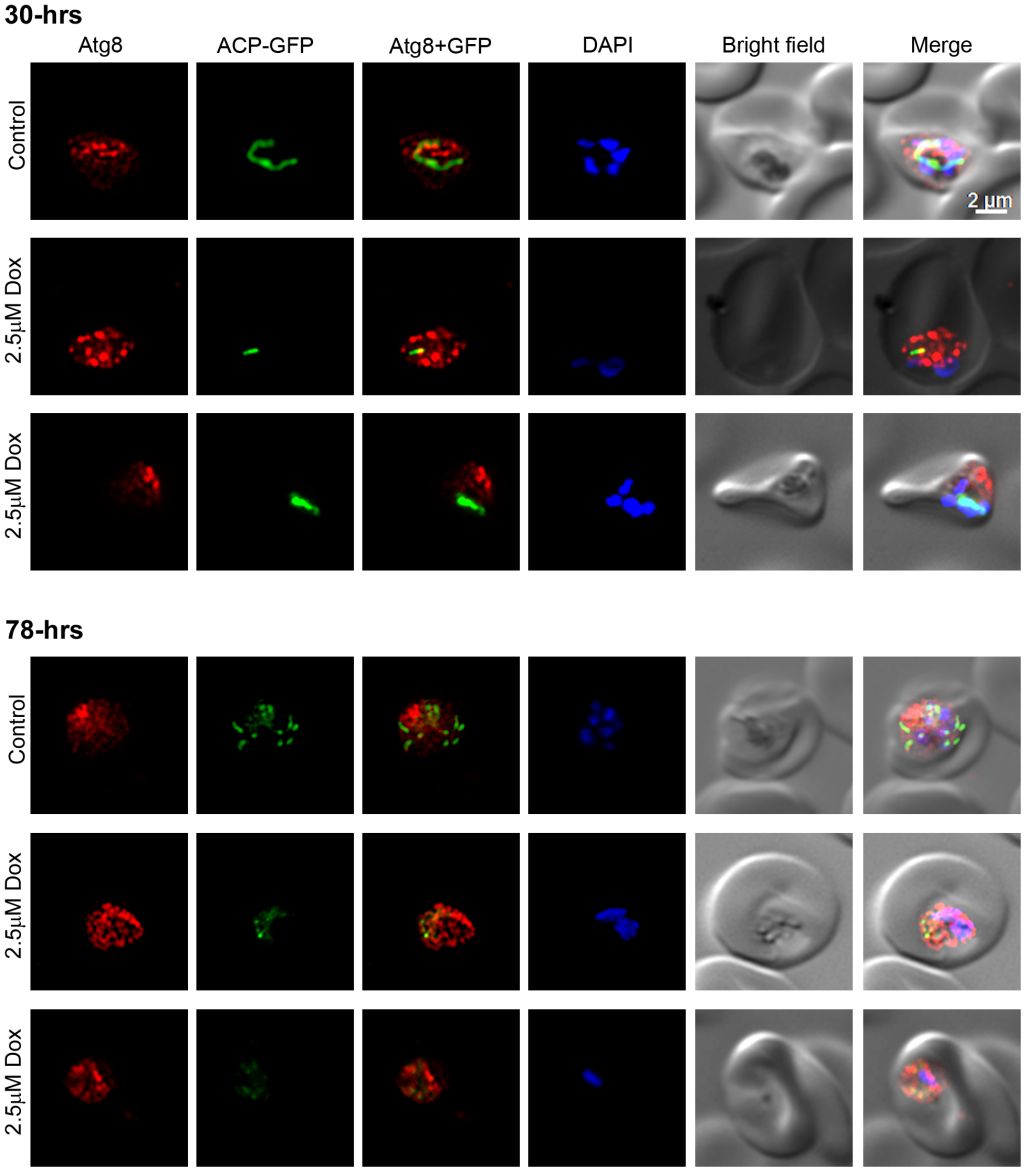
Atg8 in doxycycline-treated parasites. Synchronized ring stage *P. falciparum* D10 parasites expressing the apicoplast marker ACP-GFP were cultured in the presence of DMSO (Control) or doxycycline (2.5 µM Dox) for two full cycles, samples were collected at 30-hours (30-hrs) and 78-hours (70-hrs) time points, and processed for IFA using anti-Atg8 antibodies as described in the Materials and Methods section. The labels are as in Figure 5. The parasite images indicate that both control and treated parasites have strong signal over the elongated apicoplast at the 30-hours time point, indicating a healthy dividing apicoplast. The 78-hours control parasite have multiple intensely-stained apicoplasts, indicating that it has matured to multinucleate stage and the apicoplast has divided, whereas the treated parasites have weak fragmented signal, suggesting disruption of the apicoplast, which has been observed previously [66]. Note that Atg8 signal pattern in the control and treated parasites mostly remains unchanged.

Recently published reports have shown almost exclusive or partial colocalization of Atg8 with the apicoplast, and thereby suggested a role for Atg8 in apicoplast biogenesis [39], [40], [41], [42], [43]. We analysed the *P. falciparum* D10ACP-GFP parasites, which express an acyl carrier protein-GFP (ACP-GFP) fusion that is targeted to the apicoplast [65], for localization of PfAtg8 and the apicoplast. Anti-PfAtg8 antibodies detected punctate structures in D10ACP-GFP parasites as were also observed with 3D7 strain (Figure 5), and some of these puncta overlapped partially with the ACP-GFP signal both in dividing trophozoite and mature schizont stages. Doxycycline treatment has been shown to disrupt apicoplast biogenesis in the subsequent cycle [66]; we tested whether destruction of apicoplast affects Atg8 localization. D10ACP-GFP parasites were treated with doxycycline for two full cycles (96 hours), and evaluated for localization of Atg8 at 30 and 78 hour time points during the treatment. The treatment did not noticeably affect apicoplast in the first cycle, as both control and doxycycline-treated 30 hour time point parasites showed bright elongated apicoplast signal, but the apicoplast signal appeared weak and fragmented in 78 hour time point doxycycline-treated parasites compared to the control parasites that had progressed to late trophozoite stage and had multiple distinct bright apicoplast signal (Figure 6). However, the Atg8 localization patterns of control and treated parasites at both the time points were virtually identical (Figure 6). Our results, suggest a role of autophagy in apicoplast biogenesis. However, as the destruction of apicoplast upon doxycycline treatment did not affect Atg8 localization, autophagy function is unlikely to be exclusive to the apicoplast.

### The effects of typical autophagy inducers or inhibitors on *P. falciparum* autophagy

**Figure 7:**
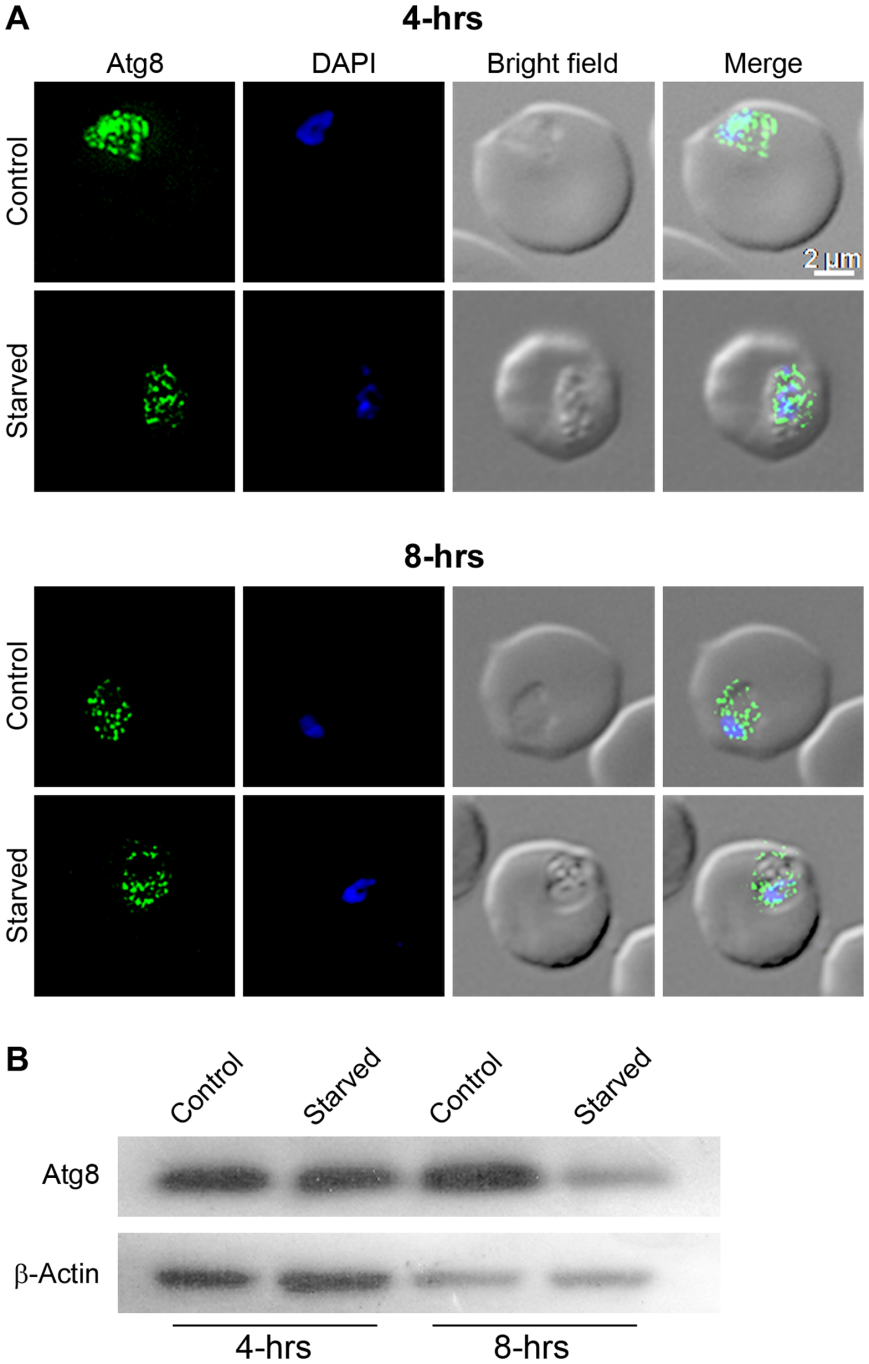
Effect of starvation on Atg8 levels and localization. The *P. falciparum* early/mid trophozoite stage parasites were cultured in complete medium (Control) or HBSS (Starved), samples were collected after four (4-hrs) and eight (8-hrs) hours of culture, and processed for localization of Atg8 by IFA (A) or expression level of Atg8 by western blotting (B) using anti-Atg8 antibodies. As a loading control, β-actin expression was also assessed in the same samples. **A**. The labels of images are as in Figure 2, and parasite images show almost similar localization patterns of Atg8 in control and starved parasites at both the time points. **B**. The western blot shows significantly reduced level of Atg8 in parasites starved for 8 hours compared to the 8 hour control parasites, suggesting downregulation of Atg8. Similar β-actin levels in control and starved parasites indicate that similar sample amounts were loaded.

Sustained supply of nutrients during starvation is probably the best-known role of autophagy. Hence, starvation is routinely used as an inducer of autophagy, which results in increased levels of Atg8-PE with a corresponding increase in the number of Atg8-labelled puncta [24], [25], [26]. We evaluated the effect of starvation on autophagy by investigating PfAtg8 localization and expression. Starvation for 4 hours did not alter localization or expression level of PfAtg8, whereas 8 hour starvation caused significant reduction in Atg8 level compared to the control parasite, but apparently did not affect the localization pattern of Atg8 (Figures 7A and 7B).

**Figure 8:**
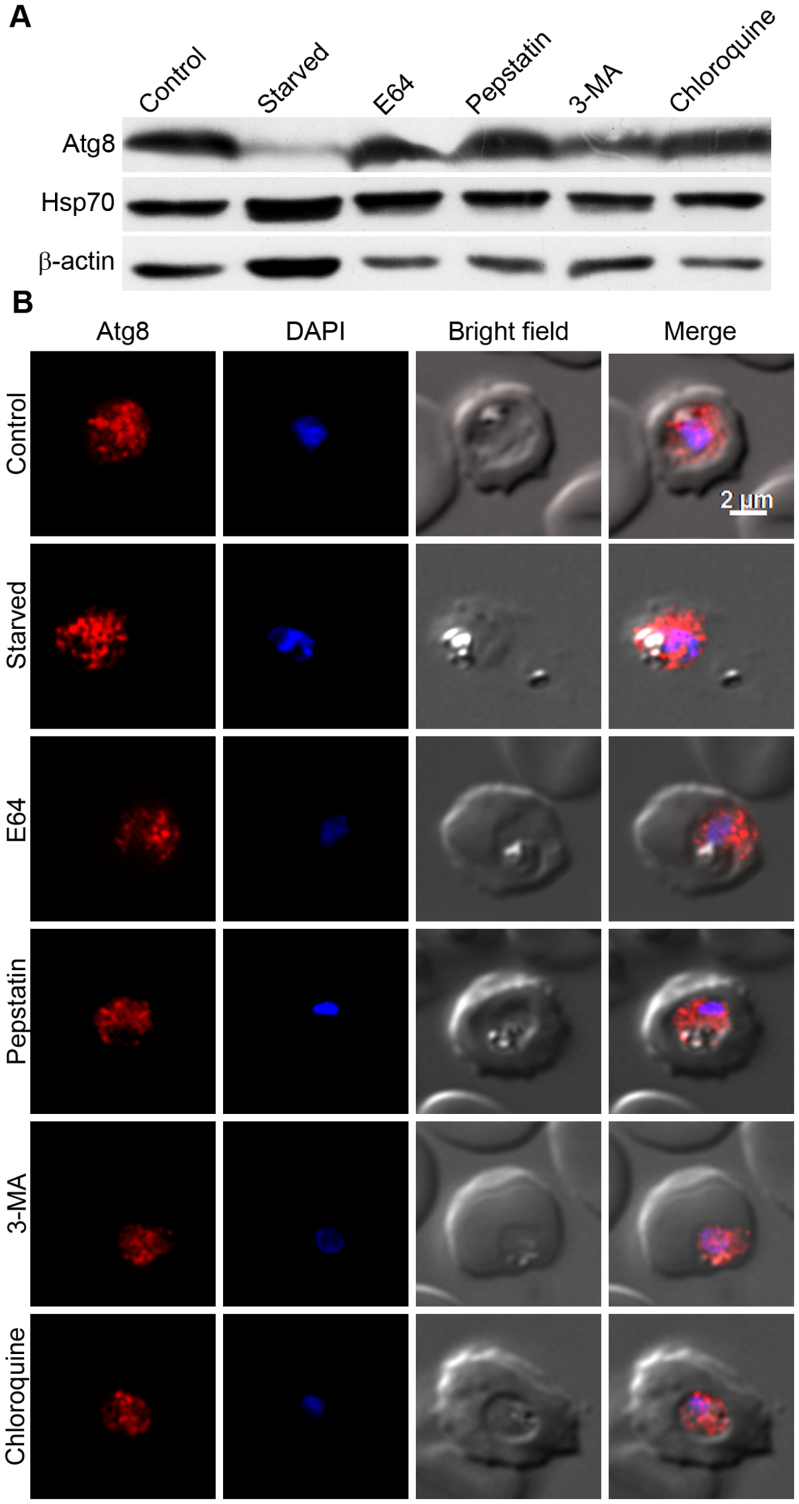
Effects of typically used autophagy inducer/inhibitors on Atg8 levels and localization. Early/mid trophozoite stage parasites were cultured in HBSS (Starved) or in complete medium containing DMSO (Control) or the indicated autophagy inhibitors (5 mM 3MA, 30 nM CQ, 22 µM E64, and 220 µM pepstatin (Pep); all except 3MA are at concentrations 3× IC_50_) for 8 hours, and then parasites were evaluated for expression of Atg8 by western blotting (A) or for localization of Atg8 by IFA (B) using anti-Atg8 antibodies as described in the Materials and Methods section. The same parasite samples were assessed for expression of the control proteins β-actin (β-Actin) and Hsp70 by western blotting as described in the Materials and Methods section. **A**. The immunoblot shows drastically reduced Atg8 levels in starved parasites and almost similar Atg8 levels in other parasite samples compared to control parasites. Both Hsp70 and β-actin levels are similar in all except the starved parasites, which may be due to a starvation-induced stress response. **B**. The parasite images are labelled as in Figure 2, and show similar Atg8 signal regardless of the treatment. The experiment was repeated three times, and the results were reproducible.

Autophagy cargo is primarily transported to the lysosomes wherein it is degraded by lysosomal proteases, which include cysteine and aspartic proteases in mammalian cells and cysteine proteases in *Leishmania* [54], [67]. Perturbing the lysosomal activity by changing its pH with the lysosomotropic agent chloroquine or by inhibiting the activity of resident cysteine and aspartic cathepsins has been shown to inhibit autophagic degradation in both yeast and mammalian cells [26], [54]. Furthermore, the kinase inhibitor 3MA has been shown to inhibit autophagy in both yeast and mammalian cells. The lysosome-equivalent organelle in malaria parasites is known as the food vacuole, and the major food vacuole proteases include cysteine proteases falcipains and aspartic proteases plasmepsins, which have crucial roles in the bulk degradation process inside this organelle, particularly degradation of haemoglobin that provides amino acids during erythrocytic stage development of the parasite [45]. To determine if autophagy has a role in nutrient supply in *Plasmodium* and/or whether falcipains and plasmepsins have a role in degradation of autophagy cargo, Atg8 localization and expression levels were assessed in starved and inhibitor-treated parasites. Trophozoite stage parasites were starved in HBSS or treated with known inhibitors of autophagy (3MA, CQ or the inhibitors of falcipains (E64) and plasmepsins (pepstatin)), and parasite samples were assessed for Atg8 expression levels and localization. Contrary to the expectation, the starved parasites had dramatically lower Atg8 level than control parasites, and treatment of parasites with protease inhibitors (E64, pepstatin) or 3MA or CQ had no significant change in the levels of Atg8 (Figure 8A). Consistent with the western blot data, the Atg8 localization patterns of treated parasite were similar to that of control parasites (Figure 8B). Virtually no effect of the above typically used autophagy inhibitors on autophagy and drastically decreased Atg8 levels upon starvation indicate an atypical autophagy in malaria parasites.

**Figure 9:**
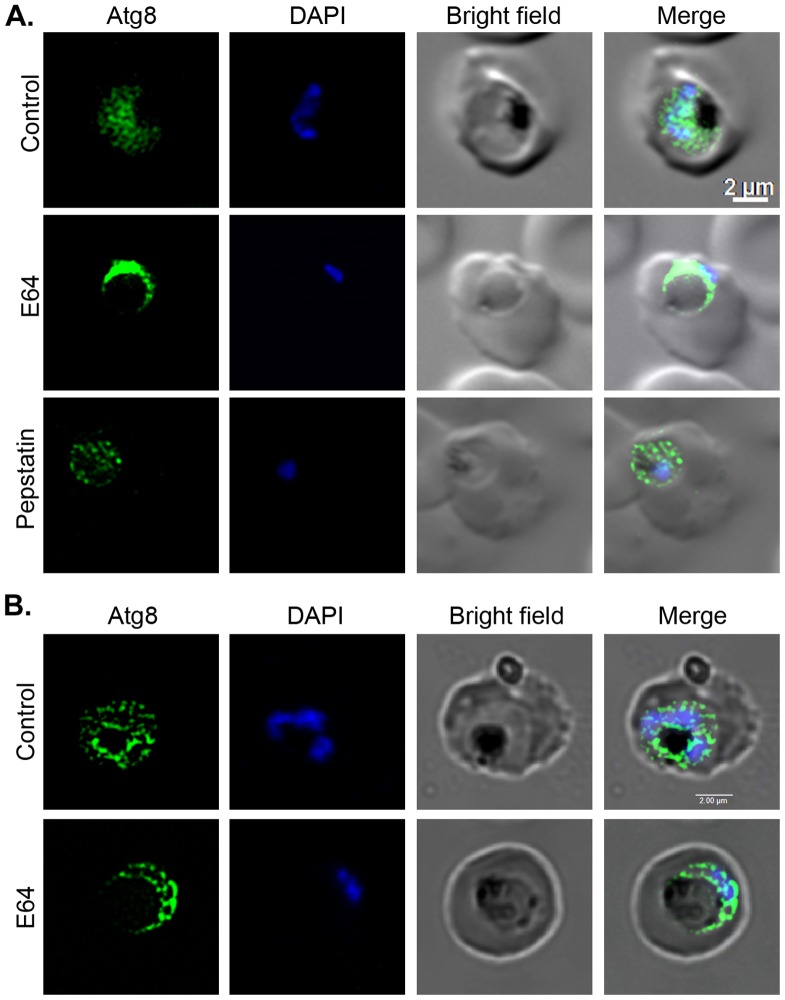
Effect of E64 and pepstatin treatments on autophagy. Synchronized early/mid trophozoite stage parasites were cultured in the presence of DMSO (Control) or inhibitors (22 µM E64, 220 µM pepstatin) for 15 hours, and then analyzed by IFA using anti-Atg8 antibodies. **A**. The parasite images are labelled as in Figure 2, and show punctate localization pattern of Atg8, which is distributed throughout the control and pepstatin-treated parasites. The E64-treated parasite show accumulation of Atg8 signal in a narrow region around the swollen food vacuole, most likely because cytoplasm has been pushed to the periphery in these parasites due to the enlarged food vacuole. **B**. Z-sections for control and E64-treated parasites were captured as described in Materials and Methods section, and the composite image shows punctate signal for Atg8 (Atg8), the stained-nucleus (DAPI), erythrocyte and parasite boundaries (Bright field), and a merge of all three images (Merge). Note that both control and E-64 treated parasites appear to have a few Atg8 puncta in the food vacuole, but not any significant accumulation of the puncta in the food vacuole of E64-treated parasites.

As mentioned above, several lines of evidence indicate crucial role of falcipains and an accessory role for plasmepsins in haemoglobin degradation, and E64 treatment is known to block falcipain activity in the food vacuole that leads to enlargement of the food vacuole due to accumulation of undegraded haemoglobin [45], [68]. As inhibition of lysosomal proteases with E64 and pepstatin in mammalian cells has been shown to cause accumulation of LC3II with increased number of the associated puncta [54], we hypothesized that inhibition of falcipains and plasmepsins by E64 and pepstatin, respectively, would result in similar effect. To test the hypothesis, synchronized 3D7 parasites at early/mid trophozoite stage were treated with E64 or pepstatin for 15 hours, and then analyzed for Atg8 localization. Parasites treated with pepstatin were arrested at the mid trophozoite stage, but those treated with E64 proceeded to the late-trophozoite stage and developed enlarged food vacuoles (Figure 9). The pepstatin-treated parasites showed punctate Atg8 signal similar to that in the control parasites (Figure 9), suggesting that plasmepsins do not have a role in turnover of autophagy cargo. The E64-treated parasites showed an intense ring of Atg8-associated puncta around the enlarged food vacuole, but the food vacuole did not have any noticeable accumulation of puncta inside (Figure 9A), suggesting that falcipains do not degrade the autophagy cargo. The Z-sections of both control and E64-treated parasites showed a few puncta inside the food vacuole (Figure 9B), which may represent the autophagy cargo for nondegradative purpose. Hence, the autophagy cargo in malaria parasites predominantly may not end up in the food vacuole, which is consistent with no effect of chloroquine on Atg8 localization pattern, and suggests that autophagy in malaria parasites is primarily not a food vacuole-dependent degradative process.

### Starvation downregulates PfAtg8 level

**Figure 10:**
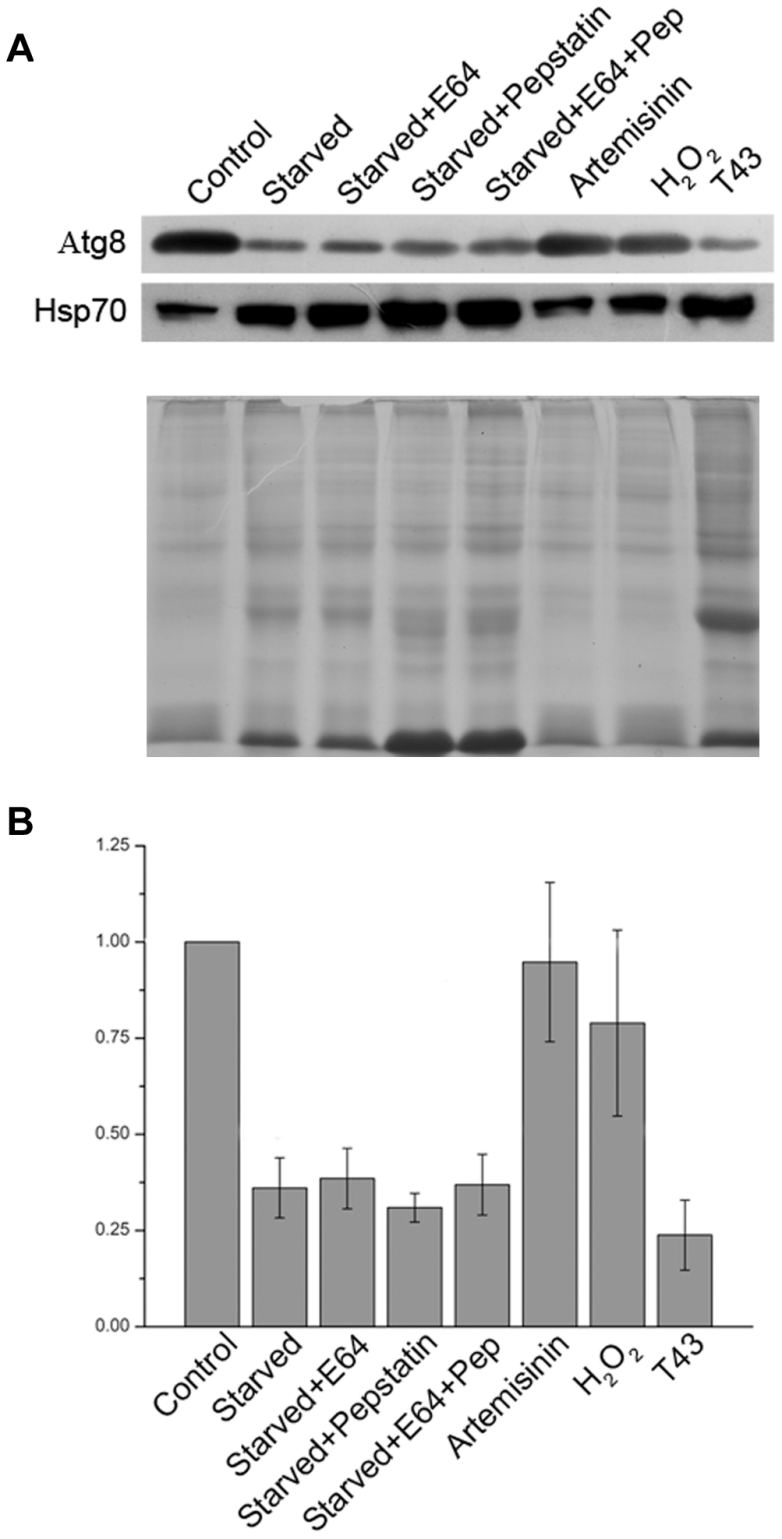
Effects of stresses on autophagy. Early/mid trophozoite stage parasites were exposed to a variety of stresses by culturing in complete medium (Control) or in HBSS containing DMSO (Starved) or the indicated inhibitors (22 µM E64 (Starved+E64), 220 µM pepstatin (Starved+pepstatin), or both (Starved+E64+Pep)) at 37°C for 8 hours. For oxidative stress, parasites were grown in complete medium containing the indicated oxidative stress-causing agents (90 nM artemisinin (Artemisinin), 100 µM H_2_O_2_ (H_2_O_2_)) at 37°C for 8 hours. For heat shock (T43), parasites were cultured in complete medium at 43°C for 8 hours. After 8 hours of the indicated treatments, equal amounts of parasite samples were evaluated for expression levels of Atg8 and Hsp70 by western blotting as described in the Materials and Methods section. Signal intensities of Atg8 in stressed parasite samples were compared with that in the control sample as mentioned in the Materials and Methods section. **A**. The blot clearly shows significantly lower Atg8 levels in starved and heat shock-stressed parasites than the control parasites; whereas artemisinin and H_2_O_2_-treated parasites have Atg8 expression levels similar to that of the control. Hsp70 expression, as expected, is significantly upregulated in starved and heat shock-stressed parasites. Notably, no effect of E64 and pepstatin alone or together on expression level of Atg8 in starved parasites suggests that downregulation of Atg8 levels is not due to degradation of Ag8 in the food vacuole. The coomassie-stained SDS-PAGE (B) below the western blot has the same sample amounts used for the western blot, which shows that sample amounts were similar across the lanes. **C**. The bar graph shows fold reduction in Atg8 expression levels in stressed parasites compared to the control parasites, and it clearly indicates almost 3-fold reduction in Atg8 levels upon starvation or heat shock. The results shown in B are means with standard deviation error bars of three western blots, which were carried out with parasite samples from two independent experiments. The experiment was repeated twice, and the results were reproducible.

The observed decrease in PfAtg8 level in starved parasites was surprising, and we investigated if it was because of increased degradation of Atg8-containing cargo in the food vacuole. Parasites were starved alone or in the presence of E64 or pepstatin or both the inhibitors together. Inhibition with either or both the inhibitors did not affect Atg8 levels, as all three parasites showed Atg8 levels similar to that in the starved only parasites (Figure 10), which was in each case almost 1/3^rd^ of the control parasites, indicating that downregulation of Atg8 level is not due to degradation of Atg8 in the food vacuole, which is consistent with the absence of Atg8 signal in the food vacuole of E64-treated parasites. As autophagy has been shown to have a protective role during stress conditions, the localization and expression level of PfAtg8 were also assessed in parasites exposed to oxidative (artemisinin and H_2_O_2_) and heat shock stresses. Atg8 level was significantly lower in parasites exposed to heat shock compared to the control parasites, but oxidative stress did not have any significant effect (Figure 10). As a control, levels of the stress response protein Hsp70 were markedly increased in starved and heat shock stressed parasites compared to the control parasites. However, intriguingly, no apparent difference in the localization pattern of Atg8 was observed in all cases (Figure S14).

## Discussion

Malaria parasites, including *P. falciparum*, seem to have limited autophagy machinery, as they contain single homologs for 11 and multiple homologs for 4 of the 33 proteins involved in autophagy in *S. cerevisiae*. The *Plasmodium* autophagy repertoire may have more than 15 proteins, as we excluded proteins that showed marginal homology with yeast Atg proteins (e-value<0.001) or lacked known conserved domains. Additionally, malaria parasites must have an ortholog of ScAtg15 for lysis of the autophagic body membrane, one or more proteases to degrade the cargo, and Atg22 to efflux degradation products. *P. falciparum* has 20 putative lipases; 11 of these are present in *P. falciparum* only and the remaining 9 are conserved. A conserved lipase (PF3D7_1427100), which shares an N-terminus hydrophobic region and a C-terminus lipase domain with ScAtg15, may function as Atg15. Cellular localization studies will be required to determine which of these are present in the food vacuole and function as Atg15. Multiple classes of proteases (falcipains, papain-like cysteine proteases; plasmepsins, aspartic proteases; falcilysin, metallo protease; dipeptidylaminopeptidase, cathepsin C-like cysteine protease; and aminopeptidases) have been shown to be present in the food vacuole wherein they have been shown or implicated in haemoglobin degradation, and these proteases may also degrade the autophagy cargo [48], [49], [51], [53], [69], [70], [71]. Several proteins are annotated as transporters on the PlasmoDB, including 5 putative amino acid transporters; cellular localization studies are necessary to determine which of these are present in the food vacuole.

Autophagy in malaria parasites seems to have two unique features. First, in contrast to the inducible nature of autophagy in *S. cerevisiae* and mammalian cells, it seems to be a constitutive process. Rather, surprisingly, nutrient starvation significantly reduced PfAtg8 level. Second, the *Plasmodium* Atg8 seems to be almost entirely membrane-associated, as all Atg8 signal was associated with punctate structures and it was exclusively present in the insoluble fraction of the parasite lysate. The lack of an ortholog of the target of rapamycin (TOR), the major negative regulator of autophagy, in malaria parasites might be a reason for constitutive autophagy. TOR is conserved in all eukaryotic organisms, including the protozoa parasites *Toxoplasma gondi* (B9PJD8), *Leishmania major* (XP_001687220), and *Trypanosoma brucei* (XP_823076). The closest TOR-related protein in *P. falciparum* is a phosphatidylinositol 3-kinase (PFE0765w, e-value = 0.00024), which has been suggested to be involved in transport of haemoglobin from the cytoplasm of infected erythrocyte to the food vacuole [55]. However, PFE0765w lacks the rapamycin-binding domain, a characteristic of TOR; hence, it is unlikely to be a TOR ortholog. Subcellular fractionation showing the exclusive presence of PfAtg8 in the insoluble fraction of the parasite lysate, consistent with the presence of almost all Atg8 signal with the puncta, further supports the constitutive nature of autophagy in malaria parasites. This could be due to ready availability of PfAtg8 for conjugation to PE, as the last carboxy-terminus residue of PfAtg8 is the conserved Gly that gets conjugated to PE. On the contrary, all characterized non *Plasmodium* Atg8 orthologs, including ScAtg8 and LC3, are first cleaved by Atg4 to produce C-terminus with exposed Gly residue, which is then conjugated to PE, forming Atg8-PE. Atg8-PE is recruited to PAS, and remains associated with the phagophore, autophagosome, and autophagic body, whereas unconjugated Atg8 is cytosolic. Similar to our data, Kitamura et al also showed that PfAtg8 is predominantly membrane-associated [39]. On the contrary, Cervantes et al showed the presence of PfAtg8 in cytosolic, nuclear, and membrane fractions [42]. This difference might be due to differences in experimental procedures used to separate the soluble and insoluble fractions in these three studies. The current study and the study by Kitamura et al used freeze-thaw method to lyse cells, which is widely used to fractionate soluble and insoluble contents of a variety of cells. On the other hand, Cervantes et al used lysis buffer that contained non-ionic detergent Igepal (0.65%), which might have extracted some PfAtg8 from membranes. Nonetheless, all three studies indicate that PfAtg8 is predominantly membrane-associated, which also agrees with its punctate localization in both asexual and sexual parasite stages.

The autophagy cargo is transported to the lysosome for degradative or non-degradative purpose. Inhibition of lysosomal protease activity by protease inhibitors or alteration in the lysosomal pH by chloroquine treatment has been shown to reduce autophagy flux that results in increased Atg8-PE levels with corresponding increase in the number of puncta, and this is considered a typical feature of autophagy [54]. In fact, perturbations in lysosomal degradation activity using inhibitors of cysteine (E64) and aspartic (pepstatin) proteases or alteration in the lysosomal pH by chloroquine has been recommended and widely used to study autophagy [25], [26]. 3MA, a kinase inhibitor that blocks autophagosome formation, is another commonly used tool for studying autophagy [24], [25], [26]. The food vacuole is the parasite's equivalent of the lysosome, and it is the site for haemoglobin degradation, a major degradative process that provides amino acids during erythrocytic stage parasite development. Falcipains and plasmepsins have been shown to be the major food vacuole proteases, and their inhibition, particularly falcipains, has been shown to block haemoglobin degradation. Hence, to determine if the autophagy cargo ends up in the food vacuole in malaria parasites, PfAtg8 expression levels and localization were evaluated in *P. falciparum* parasites treated with protease inhibitors (E64 and pepstatin), chloroquine, and 3MA. However, none of the treatments caused any significant change in PfAtg8 levels or the number of puncta. When parasites are treated with cysteine protease inhibitor E64, the food vacuole swells and is clearly visible as a large hollow under the microscope; but, we did not observe accumulation of PfAtg8-labelled vesicles in the swollen food vacuole, which indicates that majority of the autophagy cargo does not end up in the food vacuole for degradation. However, our data do not rule out the possibility of some autophagy cargo getting targeted to the food vacuole for non-degradative purpose, as both control and E64-treated parasites showed a few puncta inside the food vacuole, which is in agreement with the previously observed food vacuolar association of Atg8-associated vesicles [41]. Alternatively, other food vacuole proteases, such as dipeptidyl aminopeptidase-1 (DPAP1), falcilysin, M1-family alanyl aminopeptidase (PfA-M1), may degrade the autophagy cargo if it gets delivered to the food vacuole for degradative purpose [48], [53], [71]. No effect of 3MA and CQ on PfAtg8 expression level and the number of associated puncta in our study is in agreement with recently published reports in which another kinase inhibitor wortmannin and CQ did not have any effect on PfAtg8-labelled puncta [39], [41]. Thus, unlike in mammalian cells and yeast, no effect of E64, pepstatin, CQ, and 3MA on Atg8 turnover in *P. falciparum* shows another atypical feature of autophagy in malaria parasites, and suggests that autophagy in *Plasmodium* is primarily meant for non-degradative functions.

Another interesting feature of autophagy in *P. falciparum* is downregulation of Atg8 expression level upon nutrient starvation, which may be due to downregulation of expression or increased degradation of Atg8-coated vesicles in the food vacuole. However, as mentioned above, inhibitors of the two major classes of food vacuole proteases did not cause any noticeable change in Atg8 levels or the number of puncta, suggesting that Atg8-labeled structures are not transported to the food vacuole. This also rules out, at least to a certain extent, that decrease in Atg8 levels is not due to its degradation along with the autophagy cargo in the food vacuole. Furthermore, starvation in the presence of E64 and pepstatin did not cause accumulation of Atg8 in the parasites, and the PfAtg8 localization patterns in starved parasites and the parasites grown in rich medium were apparently similar. Thus, the decrease in levels of PfAtg8 upon starvation can be attributed to downregulation of its expression. This is in marked contrast to the situation in mammalian cells where starvation using similar conditions leads to upregulation of Atg8 levels along with an increase in the number of Atg8 puncta. As already noted, PfAtg8 is constitutively expressed and almost entirely present in membrane-associated form, it is interesting to propose that autophagy machinery in *Plasmodium* has been principally adopted for unconventional autophagy processes [3]. One reason for non-inducible autophagy during starvation could be that malaria parasites do not face starvation-like situation during erythrocytic stage development, as they are present in blood, which is rich in nutrients. Another reason for downregulation of autophagy could be the non-availability of disposable cellular contents, as the parasite has only a single organelle of each type. Hence, it could minimise the risk of accidental degradation of vital organelles by downregulating autophagy. In the context of minimising the risk of accidental degradation of vital cellular contents, it is noteworthy to mention that *Plasmodium* has been shown to enter into a hibernation state during nutrient deprivation, which has been proposed to enable the parasite to survive in malnourished host [72]. Furthermore, malaria parasites develop through a multi-stage cycle in diverse environments in which it may face inconsistent nutrient supply; the non-inducibility and downregulation of autophagy may be one of the checkpoints to minimise accidental disposal of vital cellular contents during its development.

Similar to the previous studies [41], [42], we did not observe any noticeable effect of starvation on PfAtg8 localization. However, the western blots of same parasites showed almost 3-fold less Atg8 than control parasites; the previous reports only investigated Atg8 localization, but did not determine its expression level. Since both ours and the previously reported studies showed nearly identical localization of PfAtg8 in starved and control parasites, this raises the question of how parasite can downregulate Atg8 expression level without any noticeable decrease in the number of Atg8-labled puncta. One possibility is incorporation of less number of Atg8-PE molecules in the puncta during starvation, which might not change the number of puncta drastically, as was seen in this and previous studies.

Colocalization of PfAtg8 with the apicoplast in this and the previous studies suggests a role of autophagy in apicoplast biogenesis. However, there are some notable differences between the localization patterns of Atg8 in these studies. Our data revealed the presence of Atg8-labeled structures all over the parasite, including a partial colocalization with the apicoplast; Atg8 localization appears to be almost exclusive to the apicoplast in studies by Kitamura et al and Eickel et al [39], [40], whereas Tomlins et al and Cervantes et al reported a partial colocalization of PfAtg8 and the apicoplast [41], [42]. This slight, but notable, difference in PfAtg8 localization may be due to the differences in experimental procedures used. We, Kitamura et al, and Cervantes et al used antibodies generated against the whole recombinant PfAtg8 for investigating localization of PfAtg8 in paraformaldehyde fixed cells (us and Kitamura et al) or in parasites first permeabilized with saponin and then fixed with paraformaldehyde (Cervantes et al); Tomlins et al used mCherry-PfAtg8; Eickel et al used both GFP-PbAtg8 and antibodies against a portion of PbAtg8 protein. Additionally, the parasite stage (schizonts in which the apicoplast is small vs. trophozoites that have large tubular apicoplast) used might have been responsible for differences in the observed localization patterns. We observed punctate localization of PfAtg8 throughout the parasite, including partial colocalization with the apicoplast, regardless of the parasite stage in both asexual and sexual parasites. Moreover, the localization patterns observed with anti-Atg8 antibodies (native PfAtg8 and PbAtg8) and anti-GFP antibodies (episomally expressed GFP-PfAtg8 in *P. berghei*) were similar. As doxycycline-mediated disruption of apicoplast did not cause any noticeable change in Atg8 localization, autophagy does not appear to be exclusive to the apicoplast biogenesis, which is also in agreement with the partial colocalization of Atg8 and the apicoplast.

Thus, a systematic characterization of Atg8 as a marker of autophagy in this study revealed several atypical features of autophagy in *P. falciparum*, which together suggest an atypical autophagy machinery for performing unconventional autophagy-associated functions during parasite development. Why malaria parasites evolved and what might be the functions of such autophagy are interesting questions to be addressed, but the presence of an atypical autophagy offers opportunity for developing parasite specific chemotherapeutics.

## Materials and Methods

### Materials

*P. falciparum* 3D7, D10 parasites expressing ACP-GFP (D10ACP-GFP), and *P. berghei* ANKA were obtained from the Malaria Research and Reference Reagent Resource Centre (MR4). All the biochemical reagents used in this study were from Sigma and Serva; plasmid isolation kits were from Qiagen or MACHEREY-NAGEL; cell culture reagents were from Lonza and Invitrogen; restriction and DNA modifying enzymes were from New England Biolabs; secondary antibodies and DAPI were from Invitrogen and Thermofisher. Anti-Hsp70 antibody was from Thermo Fisher Scientific (cat No. PA5-11418) and anti-β actin antibody was from Sigma (cat No. A1978-200UL). Human blood was collected from healthy volunteers after written consent under medical supervision at the medical dispensary of the institute, and the protocol (IEC-2/2010) for blood collection for this study has been approved by the Institutional Ethical Committee (IEC) of Centre for Cellular and Molecular Biology.

### Prediction of the *Plasmodium* autophagy repertoire

For identification of *Plasmodium* autophagy proteins, human and *S. cerevisiae* proteasome and autophagy protein sequences were used as queries in BLAST searches of the genomes of *P. falciparum* and other malaria parasites at PlasmoDB (http://plasmodb.org) and the National Centre for Biotechnology Information (NCBI) (http://www.ncbi.nlm.nih.gov) databases [73], [74], [75]. The identified *P. falciparum* sequences were used as queries in reverse BLAST searches against the MEROPS peptidase database (http://merops.sanger.ac.uk/), the conserved domain database (CDD) at NCBI (http://www.ncbi.nlm.nih.gov/Structure/cdd/wrpsb.cgi), and the Pfam database (http://pfam.sanger.ac.uk/) to identify conserved domains and substantiate their authenticity as autophagy proteins. The sequences were aligned using the ClustalW2 program and edited manually, and the identified *P. falciparum* proteins were annotated as homologs of the best matching human or *S. cerevisiae* autophagy proteins.

### Parasite culture

In vitro parasite culture was done according to the protocol approved by the Institutional Biosafety Committee (IBSC) of Centre for Cellular and Molecular Biology. *P. falciparum* was cultured in human erythrocytes at 2% haematocrit in the presence of a gas mixture (5% CO2, 5% O2, and 90% N2) in RPMI 1640 medium supplemented with 41.1 mg/litre hypoxanthine, 300 mg/litre glutamine, 2.5% human serum, and 0.5% Albumax II [76]. The D10 ACP-GFP strain was grown using the same culture medium with WR99210 (5 nM). Synchronization was maintained by serial treatment with 5% D-sorbitol [77]. For obtaining parasites of different developmental stages, 50 ml aliquots of a 200 ml culture at 13% parasitemia were collected at ring, early trophozoite, late trophozoite, and schizont stages. Each aliquot was centrifuged at 125×g for 5 min, the supernatant was aspirated off, and the pellet was treated with ice-cold 0.05% saponin in PBS for 5 min to lyse erythrocytes, and then centrifuged at 2450×g for 5 min at 4°C. The supernatant was discarded, the pellet was washed twice with ice-cold PBS to remove erythrocyte membranes, and parasites were recovered by centrifugation at 10000 rpm for 5 min at 4°C. The supernatant was discarded, and the parasite pellet was processed for western blot and genomic DNA (gDNA) isolation. Genomic DNA was isolated using the Puregene Blood Core Kit B (Qiagen).

For determination of IC_50_ concentrations of chloroquine and artemisinin, *P. falciparum* 3D7 strain was cultured without or with different concentrations of artemisinin and chloroquine for one full cycle, and IC_50_ concentrations of these drugs were determined as described previously [78].

*P. falciparum* gametocytes were obtained by slight modification of a previously reported procedure, which relies on induction of gametocytogenesis by spent medium [79]. Briefly, a frozen stock of the *P. falciparum* 3D7 strain was thawed and cultured in albumax-human serum medium (RPMI 1640 with 2.5% human serum, 41.1 mg/l hypoxanthine, 300 mg/l glutamine, 0.025 mg/ml gentamicin, 2 g/l NaHCO_3_, and 0.5% albumax) with erythrocytes at 2% haematocrit as described above. 20 ml of this culture (10% parasitemia with mostly rings) was centrifuged at 600×g for 5 min, and 7.5 ml of the supernatant was replaced with fresh medium (day 0); the culture was gassed, and incubated at 37°C for 24 hours with shaking at 45 rpm. On day 1, the culture was expanded to 40 ml with additional fresh medium fresh erythrocytes to adjust the haematocrit to 2%, and grown as described above. On day 2, 30 ml of the culture medium was replaced with fresh medium. On days 4 and 5, the entire culture medium was replaced with fresh medium. On day 6, the culture medium was replaced with human serum medium (RPMI 1640 containing 10% human serum, 300 mg/l glutamine, 0.025 mg/ml gentamicin, and 2 g/l NaHCO_3_). Asexual stages were eliminated by treating the parasites with pyrimethamine (200 nM) till day 9, with change of medium every day. From day 9 onwards, the culture medium was changed daily till day 14, and gametocyte development was monitored by observing Geimsa stained smears of the culture on daily basis. Gametocytes at various stages of development were collected from day 9 onwards, and processed for IFA to determine expression and localization pattern of PfAtg8 as described above.

### Production of recombinant PfAtg8

The *P. falciparum* Atg8 (PF10_0193) coding region was amplified from 3D7 gDNA using Vent DNA polymerase and primers (PfAtg8F: 5′ATG***GGATCC***CCATCGCTTAAAGACGAAGTA3′, PfAtg8R: 5′ATT***CTCGAG*** TTATCCTAGACAACTCTCACAAC3′; BamHI and XhoI sites are in bold italics; and stop codon is underlined). The PCR product was digested with BamHI and XhoI, cloned into the similarly digested pGT-GFPbsc plasmid [80] to obtain pGT-GFP/Atg8 plasmid, and the coding sequence of PfATG8 was confirmed by sequence. The pGT-GFP/Atg8 has a PstI site downstream of the XhoI site, which was used together with BamHI to excise PfAtg8. The excised fragment was cloned into the similarly digested pET-32a (Novagen) plasmid to generate pET32a-PfAtg8, which was used to transform BL21(DE3) *E. coli* cells. pET32a-PfAtg8 expresses Atg8 as a thioredoxin-His fusion (Trx-His-Atg8), which facilitates purification of the fusion protein by Ni-NTA chromatography. A culture of pET32a-PfAtg8 expression clone was induced with isopropyl β-D-thiogalactopyranoside (IPTG, 1 mM final) at OD_600_ of 0.6 for 4 hours at 37°C with shaking at 225 rpm. The bacterial pellet was solubilised in lysis buffer (8 M Urea, 50 mM NaH_2_PO_4_, 100 mM NaCl, 20 mM imidazole, pH 8.0), the lysate was centrifuged at 35000×g for 30 min, and the supernatant was incubated with Ni-NTA resin (pre-equilibrated with lysis buffer; 0.5 ml resin slurry/1.0 g weight of the bacterial pellet) for 30 min at room temperature. The suspension was transferred to a column, and unbound proteins were allowed to pass through, the resin was washed with 50× column volume of the lysis buffer (containing 20–50 mM imidazole), and the protein was eluted with 250 mM imidazole in lysis buffer. Elution fractions were run on 15% SDS-PAGE for the presence and purity of Trx-His-Atg8, and the fractions containing pure protein were pooled and dialysed against 100-fold excess of refolding buffer (20 mM Tris-Cl, 1 mM EDTA, 0.5 mM DTT, 50 mM NaCl, pH 7.5) using a 10 kDa cut-off dialysis tubing at 4°C for 16 hours with one change of the buffer after 10 hours. The dialysed protein was again dialysed against the thrombin digestion buffer (20 mM Tris-Cl, 150 mM NaCl, 2.5 mM CaCl_2_, pH 8.0), concentrated to 1 mg/ml using Ultracel-10k (Millipore) at 4°C, and digested with thrombin (250 ng/1 mg protein) at 20°C for 15 hours. Thrombin digestion of Trx-His-PfAtg8 would produce Atg8 and Trx-His. The digestion sample was incubated with Ni-NTA resin (pre-equilibrated with the digestion buffer) for 30 min at 4°C, the suspension was applied to a column, and the flow through that contained Atg8 was used in immunization and western blotting experiments, whereas the resin with bound Trx-His was discarded.

### Production of recombinant thioredoxin

The pET-32a plasmid, which has thioredoxin-His (Trx-His) coding sequence upstream of the multiple cloning site, was transformed into BL21(DE)3 cells. One thioredoxin expression clone was grown and induced as described above. The induced pellet was suspended in native buffer (50 mM NaH_2_PO_4_, 100 mM NaCl, pH 8.0; at 5 ml/g weight of the pellet), lysozyme was added (1 mg/ml final), the suspension was incubated for 30 min at 4°C, and sonicated (9 pulses on/off cycles at 20% amplitude; Sonics). The lysate was centrifuged at 35000×g for 30 min at 4°C, the supernatant was transferred to a fresh 50 ml tube, and imidazole was added (20 mM final). The supernatant was incubated with Ni-NTA resin (pre-equilibrated with the native buffer containing 20 mM imidazole; at 0.5 ml resin/g weight of the pellet) for 45 min at 4°C, applied to a column, and unbound proteins were allowed to pass through. The resin was washed with 50× column volume of the native buffer (containing 20–50 mM imidazole) to remove non-specifically bound proteins, bound proteins were eluted with 250 mM imidazole in native buffer, and elution fractions were run on a 15% SDS-PAGE for the presence and purity of Trx-His. For preparation of the Trx-His-Ni-NTA resin, bound proteins were not eluted and the resin was washed with 50× column volume of PBS, and then used for purification of anti-Atg8 antibodies as described below.

### Production of PfAtg8 antibodies

All animal experiments were carried out according to the protocol (IAEC 12/2012) approved by the Institutional Animal Ethics Committees (IAEC) of Centre for Cellular and Molecular Biology. A rabbit was immunized intraperitoneally with recombinant PfAtg8 in complete (day 0) or incomplete (days 15, 30, 60, 90, and 120) Freund's adjuvant. Serum was collected on days 75, 105, and 135 and used for determining reactivity against recombinant PfAtg8 by western blot as described below. Final serum was collected on day 140, and it was fractionated using ammonium sulphate method to obtain IgGs [81]. The IgGs solution (in PBS) was incubated with the Trx-His-Ni-NTA resin and then with fixed BL21(DE3) cells to remove any cross-reactive antibodies. The purified anti-Atg8 sample was supplemented with sodium azide (0.2%), filtered through a 0.2 µm filter, and stored at −20°C for use in western blotting and IFA experiments. To evaluate reactivity and specificity of anti-Atg8 antibodies, recombinant and native Atg8, recombinant intact Trx-His-PfAtg8 fusion (2 µg), purified PfAtg8 after thrombin digestion (2 µg), uninfected red blood cell lysate, and parasite lysates (corresponding to approximately 1×10^8^ mixed stage parasites) were run on a 15% SDS-PAGE gel, transferred onto the Immobilon-P membrane, and probed with purified rabbit anti-PfAtg8 (at 1/1000 dilution) followed by with HRP-conjugated goat anti-rabbit IgG (Invitrogen; at 1/10000 dilution). The signal was developed with the SuperSignal West Pico Chemiluminescent kit (Thermo Fisher Scientific) on X-ray film.

### Western blot assay

To determine expression of Atg8 in ring, trophozoite, and schizont stages, parasite pellets were suspended in 10 mM Tris, pH 7.5 and equal volume of 2× SDS-PAGE sample buffer (1× buffer contains 62.5 mM Tris-HCl, 20% glycerol, 2% SDS, 1% β-mercapto ethanol, 0.01% bromophenol blue, pH 6.8) was added to the lysate, and the sample was sonicated (3 pulses of 15 seconds each at 20% amplitude). The sample was incubated at 100°C for 10 min, centrifuged at 24000×g for 20 min, and supernatants were transferred to fresh tubes and used for western blot. The supernatants of parasite lysates (corresponding to approximately 1×10^8^ parasites) were run on a 10% SDS-PAGE gels, and transferred onto the Immobilon-P membrane. The membranes were treated with 1% paraformaldehyde [82], blocked with blocking buffer (5% milk in PBS-Tween 20) for two hours at room temperature, incubated with primary antibodies (rabbit anti-Atg8 at 1/1000, rabbit anti-Hsp70 and mouse anti-β-actin at 1/500), in blocking buffer for overnight at 4°C. The blots were washed with the blocking buffer, incubated with secondary antibodies (HRP-conjugated goat anti-rabbit and HRP-conjugated goat anti-mouse IgGs were used at a dilution of 1/10,000 in blocking buffer) for 1 hour at room temperature, and signal was developed with the SuperSignal West Femto Chemiluminescent kit (Thermo Fisher Scientific) on X-ray film.

### Expression and localization of PfAtg8 in asexual and sexual *P. falciparum* stages

For expression and localization of PfAtg8 in asexual and sexual stages, parasites at different stages of development were analysed by IFA. Cells were washed with PBS, layered on a poly L-Lysine coated slide for 20 minutes, unbound cells were washed off with PBS, and the immobilized cells were fixed (3% paraformaldehyde and 0.01% glutaraldehyde) for 1 hour. The cells were permeabilized with 0.1% Triton X-100 (in PBS) for 30 minutes, blocked with blocking buffer (3% BSA in PBS) for overnight at room temperature, and incubated with anti-Atg8 antibodies (1/50 in 3% BSA) for 1 hour. The cells were washed with blocking buffer, incubated with secondary antibody (Alexa Fluor 488 donkey anti-rabbit IgG or Alexa Fluor 594 donkey anti-rabbit IgG at 1/2000 dilution in 3% BSA) for 1 hour, and then incubated with the nuclear stain DAPI for 20 minutes (10 µg/ml in PBS). The slides were air-dried, mounted with ProLong Gold anti-fade reagent, the sample area was covered with a coverslip, and the edges were sealed with nail polish. The slide was observed under 100× objective of the AxioImager.Z1 with Apotome, images were captured with AxioCam, and analysed using the AxioVision LE software.

### Subcellular fractionation

A 50 ml culture of *P. falciparum* 3D7 trophozoite/schizont stage parasites at 20% parasitemia was harvested to isolate parasites as described above. The parasite pellet was suspended in 400 µl PBS containing protease inhibitor cocktail (ProteCEASE-50, G-Biosciences), and subjected to 5 cycles of freezing at −80°C and thawing at 37°C. A fraction of the lysate was taken aside as the total lysate. The remaining lysate was centrifuged at 200,000×g for 1 hour at 4°C. The supernatant, which would contain soluble cytosolic proteins, was transferred to a fresh tube. The pellet was washed with PBS (with PIC) and divided into three equal parts. One part was taken aside for the freeze-thaw pellet fraction, and resuspended in PBS. The other two parts were resuspended in peripheral membrane extraction buffer (100 mM Na_2_CO_3_, pH 11.5) or integral membrane protein extraction buffer (2% Triton X-100 in PBS), and incubated for 2 hours on ice. The suspensions were centrifuged at 200,000×g for 1 hour at 4°C, and supernatants were transferred to fresh tubes, and the remaining pellets were resuspended in their respective buffers. 2× SDS-PAGE sample buffer was added to the lysate, incubated at 100°C for 10 minutes, centrifuged at 20,000×g for 20 minutes and the supernatants were transferred to a fresh tube. All the fractions were normalized to equivalent number of cells (2×10^7^ parasites/µl) and equivalent amounts of all samples were evaluated for the presence of PfAtg8, β-actin, α2 subunit of the proteasome by western blotting. Rabbit anti-α2 subunit primary antibodies (1/500 dilution) and mouse anti β-actin (1/500 dilution) were used as primary antibodies, and anti-mouse HRP and anti-rabbit antibodies were used as secondary antibodies at 1/10000 dilution. Blots were developed as described earlier.

### Episomal expression of Atg8 in *P. berghei*

*P. falciparum* Atg8 was amplified from PfgDNA (primers: PfAtg8F, 5′-ATGGGATCCCCATCGCTTAAAGACGAAGTA-3′; PfAtg8R: 5′-ATTCTCGAGTTATCCTAGACAACTCTCACAAC-3′), the PCR fragment was cloned into the pGT-GFPBsc plasmid [80] at the BamHI-XhoI site downstream of GFP, and the sequence was confirmed. The GFP-PfAtg8 fusion was excised with NcoI (internal site in GFP) and XhoI, and ligated with the similarly digested pSTCII-GFP plasmid [80] to construct the pSTCII-GFP-PfAtg8 transfection plasmid.

Transfections in *P. berghei* ANKA were done essentially as described previously [80], [83]. BALB/c mice were infected with a frozen stock of *P. berghei* ANKA and parasitemia was monitored by making smears of the blood from the tail snips. Mice were sacrificed at 5–6% parasitemia, blood was collected in Alsevier's solution by cardiac puncture, and parasites were cultured in RPMI-FBS medium (RPMI 1640 with 20% FBS) for 12–14 hours at 34°C with gentle shaking and periodically monitored the development by making giemsa smears. When majority of the parasites matured to schizonts, parasites were isolated by purification on 60% Histodenz gradient (Sigma). The purified schizonts were suspended in 100 µl transfection reagent (T cell Nucleofector transfection kit, Lonza), circular pSTCII-GFP-PfAtg8 plasmid (5 µg DNA) was added, and the contents were transferred to the supplied cuvette and electroporated using the program U-033 (Amaxa Nucleofector device). 100 µl culture medium was added to the cuvette, and the entire sample was injected intravenously into a naive mouse. The mouse was given pyrimethamine in drinking water (70 µg/ml) for selection of recombinant parasites. Resistant parasites appeared after 7–9 days, which were analysed for expression of GFP-PfAtg8 by IFA as described below.

### Localization of native and episomally-expressed Atg8 in *P. berghei*

To determine expression and localization of native PbAtg8 in *P. berghei*, 5–10 µl blood was collected from tail snips of BALB/c mice infected with wild type *P. berghei* ANKA parasites, cells were incubated with anti-PfAtg8 antibodies (1/50) followed by Alexa fluor 488 donkey anti-rabbit antibodies (1/2000) and DAPI as described above.

For localization of episomally expressed GFP-PfAtg8, 5–20 µl blood was collected from the tail snips of mice infected with recombinant parasites. The cells were incubated with rabbit polyclonal anti-GFP antibodies (1/1000), followed by Alexa fluor 488 donkey anti-rabbit antibodies (1/2000 dilution) and DAPI. For colocalization studies, parasites expressing GFP-PfAtg8 were incubated sequentially with goat polyclonal anti-GFP antibodies (1/1000), DyLight 594 rabbit anti-goat antibodies (1/2000 dilution), anti-Atg8 antibodies, followed by Alexa fluor 488 donkey anti-rabbit antibodies (1/2000) and DAPI. Images were captured using AxioImager.Z1 with Apotome.

### Colocalization of Atg8 with the apicoplast

For co-localization studies of Atg8 and apicoplast, *P. falciparum* D10 parasites expressing ACP-GFP (D10ACP-GFP) were collected at different asexual stages of development. Parasites were fixed (3% formaldehyde and 0.01% glutaraldehyde), incubated with anti-Atg8 antibodies, and then with Alexa Fluor 594 donkey anti-rabbit secondary antibodies as described above.

For doxycycline treatment, a synchronous culture of D10ACP-GFP parasites was grown in the presence of DMSO (0.1%) or 2.5 µM doxycycline (5 times of IC_50_ concentration [66]) for two cycles, with change of medium after the first cycle. Parasites were collected at 30-hour and 78-hour time points, and analyzed for localization of Atg8 and apicoplast by IFA as described above.

### Inhibition and starvation assays

Synchronous cultures of *P. falciparum* 3D7 parasites at early/mid trophozoite stage (10% parasitemia) were grown in Hank's Balanced Salt Solution (HBSS) (starvation) or in the complete culture medium for 4 or 8 hours. To investigate the effects of various inhibitors or starvation or heat stress, early/mid trophozoite stage parasites (10% parasitemia) were cultured in HBSS (with or without E64 or pepstatin) or in the complete medium with 0.45% DMSO (control) or with inhibitors (5 mM 3MA), 22 µM E64, 220 µM pepstatin, 30 nM chloroquine, 90 nM artemisinin; all concentrations except 3MA are 3 times of the IC_50_ [78]) or H_2_O_2_ (100 µM) at 37°C or at 43°C (heat shock stress) for 8 hours. In all cases, parasites were collected after the treatment, evaluated for localization of PfAtg8 by IFA as described above, and for expression levels of PfAtg8, Hsp70 and β-actin by western blot. For western blot, parasites were isolated, lysates were prepared and similar amounts of the lysates were run in 10% SDS-PAGE, and transferred to the nitrocellulose membrane as described above. The membranes were incubated with rabbit anti-Atg8 (1/1000 dilution) or rabbit anti-Hsp70 (1/500 dilution) antibodies, followed by secondary antibodies (HRP-conjugated goat anti-rabbit antibody at 1/10,000 dilution). The blots were developed as described above, and the signal intensities were measured by densitometry using the software ImageJ, and plotted using the GraphPad Prism software.

To investigate the effect of prolonged inhibition of the food vacuole protease activity on Atg8 localization, synchronized 3D7 parasites at early-mid trophozoite stage were treated with E64 (22 µM) or pepstatin (220 µM) for 15 hours, and then analyzed by IFA using anti-Atg8 antibodies. Images were captured using AxioImager.Z1 with Apotome. Z-sections for control and E64-treated parasites were captured using Leica TCS SP8 confocal laser scanning microscope and edited using the Leica Application Suite software.

## Supporting Information

Figure S1 **Alignment of the Atg11 proteins.**
(PDF)

Figure S2 **Alignment of Vps34 proteins.**
(PDF)

Figure S3 **Alignment of Vps15 proteins.**
(PDF)

Figure S4 **Alignment of the Atg18 proteins.**
(PDF)

Figure S5 **Alignment of Atg8 proteins.**
(PDF)

Figure S6 **Alignment of Atg4 proteins.**
(PDF)

Figure S7 **Alignment of Atg7 proteins.**
(PDF)

Figure S8 **Sequence alignment of Atg3 proteins.**
(PDF)

Figure S9 **Sequence alignment of Atg12 proteins.**
(PDF)

Figure S10 **Sequence alignment of Atg5 proteins.**
(PDF)

Figure S11 **Alignment of the Atg23 proteins.**
(PDF)

Figure S12 **Expression of recombinant PfAtg8.**
(PDF)

Figure S13 **Expression and localization of Atg8 in sexual erythrocytic stages.**
(PDF)

Figure S14 **Effects of stresses on autophagy.**
(PDF)

Table S1 **Identity of** ***P. falciparum*** **Atg proteins with other** ***Plasmodium*** **homologs.**
(PDF)
