# Supplementary material for: Characterization of the Autophagy Marker Protein Atg8 Reveals Atypical Features of Autophagy in Plasmodium falciparum
Source: PLoS One. 2014 Nov 26;9(11):e113220. doi: 10.1371/journal.pone.0113220 (PMC4245143; doi:10.1371/journal.pone.0113220)
Supplement: Figure S1 — Alignment of the Atg11 proteins. (PDF) [file pone.0113220.s001.pdf]

```
ScAtg11          ---------------MADADEYSTAPTQQEITPLQTTATIINAISGECITTNVDF---FV 42
PfAtg11          YTKNKSDLFNNYNLNESLNDSYKDKTFEELKSQLIKQKNLNSCLSLKLKSIHIKLNNLFL 60

ScAtg11          SLDKFKQFIARKWKIPPDQLLILLPYGNKLKPSMFKELLINRSFTLN----DFYVYDRRL 98
PfAtg11          KKNEFEKTINNKIKEIELQFLIIQNVTHENKGIPISKELKEQEKHLQNSHENVAIYDTHE 120

ScAtg11          FSLVSKPTPTNLLTSKDSNPMNSPNSNDLTETLEYLIKNSHISQYQGSDTIMIKPMPSPL 158
PfAtg11          IENNDKKKLYTNFHNEEKDHLKCLLEEYSKTLEIYKMGKIQLEFELKCCKEKLNEEIEKN 180

ScAtg11          EDADVDLS--RLNYHSVTSLLTTNLGWLSALEIDVHYFKS----------LIPDIIAHIK 206
PfAtg11          NNYNNKMKSYEIHIDVVKNENCKNLEELNDLKLQLEKTKSENNQNYVKNKILNDEKNNLD 240

ScAtg11          RIFDGLTVCSQYLKLYCFDVES-------------------LYNSNVQFLNQLVDNGMTS 247
PfAtg11          KINNDLKIKIKNFKTLLNDAQNKEYILNNFTQKIFNIITYLKNNDEHNFLNDKIHNKLDI 300

ScAtg11          KWEKCFNDTLSKLTALEGDSLQKFINIESLLEN-----EKSVKILNHSINGKLNKIKREI 302
PfAtg11          NQDQIYVQTELYIDIIS-SSIRNLINFKKTLEERNVELEKVTHEMKELRKELILKKKNYE 359

ScAtg11          DENASFRDIITVNIDRLRQMFTPNESK---FELEDQMAESFEVLVSEMRTRSRNVLDKEE 359
PfAtg11          ELRLKLNHLECVERDSVKINSEKEKGEKVIYELKEKLDNDEKIINDLKKKNSYQVYKMKD 419

ScAtg11          EEFNSQEFLKSMN---VMLEKD----------KKESVKTLFTISQALYSQIGELIDLKKS 406
PfAtg11          YEKRENNLINEINKLKLFIEENKMTVERGNEMNNKKLEEMKQKNKELINNLNDISDELKN 479

ScAtg11          LQKHAVAILGNIAFTQMEILGIKRLLLNECNKDLELYKKYEVEFAQVEDLPLIYGLYLIE 466
PfAtg11          CIEQVNSVSRNMANVEKEKENIINELQILRMKNDTMRKRISKFVEQEKVLKFKLYTLNND 539

ScAtg11          KYRRLSWFQQILSFISNFNQDLELFKQNELRTRNKWVKNFGSIATVFCEDLLSSSDFKRL 526
PfAtg11          IFSKNEKLNDMQKKLNDVNEKYKNIVECLNNYKTEHKEQIEKKIERINTLKQNYYYLKKE 599

ScAtg11          NEYHSHTSPPNEDEEDENENSIANYRQDLVKVSQAIDNYMTQIKETDVSEPIIDLLSKTL 586
PfAtg11          YDLKNKELEKNIEHGKKLEHELSHCYEENQKLNEEIKRRNSFIKNKDRKIDLLTNIENEL 659

ScAtg11          FETKRFHIIYSNFKN---NNNNSSNGNSISPEGSIALKSDDVVKGYKTRIKKLESLLHEF 643
PfAtg11          LKKKEINNIKLMEKQNVIKNNEQLLKDIKDENEKMNEHVNKLQNELIKRELQNKCISKDI 719

ScAtg11          QYS------DIGHWPQGVLN--THLKPFRGSATSINK----KKFLGASVLLEPANISEVN 691
PfAtg11          EFCKKEKEDKIKNLEDDLLEKKKCIENLKDELINIKKKMEDKMHMTNEMDLLSNKVEELN 779

ScAtg11          -IDSVSQANNHQIQELESNVDDLLHQLQLLKEENNRKSMQISEMGKKISDLEVEKTAYRE 750
PfAtg11          RINKTYEKNIVELNNELDVIKKKLNDEEFLKEEEKKKNIDMVYKIKEYEIQIKEKENEID 839

ScAtg11          TLTNLNQELARLTNEEQSHRTEIFTLNASFKKQLNDIISQDNEKIEK----LTGDYDDVS 806
PfAtg11          SLKKNEQNLHVLKNEELNEKEIILKN--KYDKEINMIIEQYNKKIQEEKDMLNNKIKSMD 897

ScAtg11          KSRERLQMDLDESNKKHEQEVN-----------LLKADIERLGKQIVTSEKSYAETNSSS 855
PfAtg11          QTHKNQIEEMQEENKKELKRLKNVCDMNLQSQILIKENEKHMQEKVEEYKNLLKQKDQEL 957

ScAtg11          MEKGEKFETIPLAEDPGRENQISAYTQTLQDRIFD----IISTNIFILENIGLLLTFDNN 911
PfAtg11          KNIIQEYDERIEIQNKEMEDIVNDCEEKLKQAKINNKKLTTATNMANNNNMLMDENLKEK 1017
```

Figure S1. Alignment of the yeast Atg11 (ScAtg11) and its putative *P. falciparum* homolog (PfAtg11) was performed using the Clustal W2 program. Shown are conserved residues (in red).
