# Supplementary material for: Characterization of the Autophagy Marker Protein Atg8 Reveals Atypical Features of Autophagy in Plasmodium falciparum
Source: PLoS One. 2014 Nov 26;9(11):e113220. doi: 10.1371/journal.pone.0113220 (PMC4245143; doi:10.1371/journal.pone.0113220)
Supplement: Figure S2 — Alignment of Vps34 proteins. (PDF) [file pone.0113220.s002.pdf]

```
PfVps34         mkirydkcsstkdlnyffhlklgffvcyknhndkysfknkilqkndtilffkkkkkfmyl 60
PfVps34         rkkkkkkkkkiliqiiqeynkyneyfkynsnlegnqgfnkkpeknkntkgnvytdhtnqn 120
PfVps34         akskiynydmnddsysnyvnnnnvfrissflilnneffgyplqfvcetegrsrnhehypd 180
PfVps34         vhgdnikynkcddnkynkcddnkydkcddnkynkcddnkydtcddnkydtcddnkydtcd 240
PfVps34         dnkynkydddkydtcddnkynkydddkynkydddkynkydddkyeksrkkkklnnlykti 300
PfVps34         ltkkkrkkmnsnlcvinkiykypikycelnskafvffiiknvgvhkityysynklfskdg 360
PfVps34         vlnqgiqicklyhvnknkkikqiifealknkitfsydnnpnnikkkiykflkkncayhdl 420
PfVps34         iklfyfkghkqrekcnkklnmektfgvhkssrynyktykkkkkidmcknycddildtyns 480

ScVps34         ----------------------------------------------------MSLNNITF 8
PfVps34         kyykgelsgqhkhikmtgeqkeehhikythlnfnhgkdetfykelykcnyieKYISSVNY 540

ScVps34         CVSQDLDVPLKVKIKSLEGHKPLLKPSQK------------------------------- 37
PfVps34         FLLERRRMFNKYKQQELCVNKNEENNKNKNDDDNKNDDDNKNDDDNNKNDDDNKNDDDDN 600

ScVps34         --------------------------------------------ILNPELMLIGSNVFPS 53
PfVps34         KNDDDNKNDDDDDNKNDDDDNNKNNIQCDNHSDNIYMCGTYGNMENYNVPHSTNNTNLQSI 660

ScVps34         SDLIVSLQVFDKERNRNLTLPIYTPYIPFRNSRTWDYWLTLPIRIKQLTFSSHLR----- 108
PfVps34         KKRIINMNILDNIRCNKTYKYIDKNKFKCFTYYSCKNYNVCKKIIEKYKLYKFLKKKKIE 720

ScVps34         --IILWEYNGSKQIPFF------------------------------------------- 123
PfVps34         GYMILNFLNFNKELIYYNEHKKDMSTLHDNLFDVISNNQNENVKYNHICNNNKYDWFFNS 780

ScVps34         -----NLETSIFNLKDCTLKRGFESLK------FRYDVIDHCEVVTDNKDQENLNKYFQG 172
PfVps34         FDYVGNLEESITCFNNHKKKENMKNIKNIKKKKKKNLFYNEQHNIKNNKNDYHFDKYPSS 840

ScVps34         EFTRLPWLDEITISKLRKQREN-------------------------------------- 194
PfVps34         LYSHLTNKKMVNNTEVNNIKDENSLQMYIINKDVTKNKDGNLLLNSYYNSKLGKSINTCS 900

ScVps34         ----RTWPQGTFVLNLEFPMLELPVVFIEREIMN-------------------------- 224
PfVps34         KEIYKEEHKNVYIYNKKITKMNIKMKTEQKYICVDSKRNTRTYNSKNIRTYNSKNIRTYN 960

ScVps34         -------TQMNIPTLK-------NNPGLSTDLREPNRNDPQIKISLG-----DKYHSTLK 265
PfVps34         SKNIRTYNRKNIRTYNRKNIRTYNRKNIRTYNRKNIRCNNRKKFHLNRNKKKNGCVKKYK 1020

ScVps34         FYD------------------------PDQPNNDPIEEKYRRLERASKNANLDKQVKPDI 301
PfVps34         LYDERNTLVYKNKIGSNHFFLKEEIGKSTKKLNDIFEHISNYTNRISKNINITNKNRYDD 1080

ScVps34         KKRDYLN--------------------KIINYPPGTKLTAHEKGSIWKYRYYLMNNKKAL 341
PfVps34         YPFDFLSKDKIEYISMLSPTINEIKTLNTILTIPLIKMNEYEKNCIWRFRFQLLNRKETL 1140

ScVps34         TKLLQSTNLREESERVEVLELMDSWAEIDIDDALELLGSTFKNLSVRSYAVNRLKKASDK 401
PfVps34         GKFLKSINWNNKEEEEEAIILLNKWAKPGIENCIELFYSHLHHYVIKKYIIDIIKNSKKE 1200

ScVps34         ELELYLLQLVEAVCFENLSTFS-------------------------------------- 423
PfVps34         EIKLYLFQLVQSLRTFNYQHIDNLFINTLIQKCIKSKKLSIYFYWFLLSEAKDKIKGKLY 1260

ScVps34         -------------DKSNSEFTIVDAVSSQKLSGDSMLLSTSHAN---------------- 454
PfVps34         LHIHKLFINKLMTSNIRKNKIILDILKNQNRFRNQLLYLTKIAKNKTDRIQNKTRKLRNF 1320

PfVps34         lfyyrtnygyinikdfiknnifisdhnvydfldickmkrensldtpmrgdnigqpsylgm 1380
PfVps34         vpgmgkstddsknvygddnknvygddnknvygddskniycddnknvygddnkniygddsk 1440

ScVps34         ------------------------------QKLLKSISSESE---------TSGTESLPI 475
PfVps34         niygddnknifsddnknlysdnnnnkhiryNKYVKNISYEHFNEYPYDNKKSRNIYTCNK 1500

ScVps34         VISPLAEFLIRRALVNPRLGSFFYWYLKSESEDKPYLDQILSS------FWSRLDKKSRN 529
PfVps34         DICNSIYYLDNELTINYDIKDDLYFFQYKRSSDEKLLNTDLSNDSNDMIHYIDDSKNVKI 1560

ScVps34         ILNDQVRLINVLRECCETIKRLKDTTAKKMELLVHLLETKVRPLVK------VRPIALPL 583
PfVps34         ERNRDNSFFSNFLQFNDNLDFFLNATYSDEDNNYEILDDSINFVQKQKIKKIKTPLILPI 1620

ScVps34         DPDVLICDVCPETSKVFKSSLS-------------------------------------- 605
PfVps34         DPNIELLSFLPEQSYVLRSSLYPIVIACLVRKKIKLYNENYNNLIINNHTFYKNDQNKDN 1680


ScVps34         ---------------------PLKITFKTTLNQPYHL----------------------- 621
```

```
PfVps34         IINNLSYDKSYHSYYNSQFIKTLQNSFESTTSLNYHYNFLKCSNNNIFYKNKKIERIKPN 1740

PfVps34         tsiqkafpsnenilnrnqhvyysnnqivhnikkmnkhkrddyminekvlpcvsnsclgdk 1800
PfVps34         lmpshdkmrsshdkmmpshdkmmpshdklmsphytlmsshdkpvapsgvsslgekkskde 1860

ScVps34         ------------------MFKVGDDLRQDQLVVQIISLMNELLKNENVDLKLTPYKILAT 663
PfVps34         KKNRKKYNEIYQLSIKKYIYKAGDDLRQDHLVIQIIYVMDNIWKRYGLDLKMTLYRVLAL 1920

ScVps34         GPQEGAIEFIPNDTLASILSKYHG--ILGYLKLHYPDENATLGVQGWVLDNFVKSCAGYC 721
PfVps34         STDDGFIEFVDYAESISSIKKNYKGEIRQYFIDNSTCSSSPLGFDTEILQNFISSCAGYS 1980

ScVps34         VITYILGVGDRHLDNLLVTPDGHFFHADFGYILGQDPKPFPPLMKLPPQIIEAFGGAESS 781
PfVps34         VITYILGIGDRHLDNLMVTKDGRFFHIDFGYIFGEDPKPFSPPMKLCKEMIEAMGGAHSI 2040

ScVps34         NYDKFRSYCFVAYSILRRNAGLILNLFELMKTSNIPDIRIDPNGAILRVRERFNLNMSEE 841
PfVps34         GYEQFLKKCCLAYKYLRYHSQLIISLLDAMCDAGLKDMKMSPELCVLKVQEKFRLDLNDE 2100

ScVps34         DATVHFQNLINDSVNALLPIVIDHLHNLAQYWRT 875
PfVps34         AAEIYFLSVINASVKTLFPVVVDKLHEWALNWK- 2133
```

Figure S2. Sequence alignment of Vps34 proteins. The yeast (ScVps34) and *P. falciparum* (PfVps34) Vps34 protein sequences were aligned using the Clustal W2 program. Shown are the conserved residues (red), the putative N-terminal lipid binding C2 domain of the ScVps34 (underlined), phosphatidylinositol 3-phosphate kinase accessory (PI3Ka) domain of unknown function (yellow shaded), phosphatidylinositol 3-kinase domain (grey shaded bold letters), and PfVsp34-specific large inserts (lower case).
