# Supplementary material for: Characterization of the Autophagy Marker Protein Atg8 Reveals Atypical Features of Autophagy in Plasmodium falciparum
Source: PLoS One. 2014 Nov 26;9(11):e113220. doi: 10.1371/journal.pone.0113220 (PMC4245143; doi:10.1371/journal.pone.0113220)
Supplement: Figure S3 — Alignment of Vps15 proteins. (PDF) [file pone.0113220.s003.pdf]

```
ScVps15          MGAQLSLVVQASP-------------SIAIFSYIDVLEEVHYVSQLNSSRFLKTCKALDP 47
PfVps15          MGNTLYSNIGHSTTELDDVYCKYLNKLFNIYNYLFKYEHFFFMNSYTLNNFYHVLEGINN 60

ScVps15          NGEIVIKVFIKPKDQYSLRPFLQRIRAQSFKLGQLPHVLNYSKLIETNRAGYMIRQHLKN 107
PfVps15          NEGHVLIKICKLKSEAPKIKRILYTLKFLFSFDLFPNVLPYNRMSVYENNIYIYRRFIFK 120

ScVps15          NLYDRLSLRPYLQDIELKFIAFQLLNTLKDIHNLNIVHGDIKTENILVTSWNWCILTDFA 167
PfVps15          NLDHYLSNKINTNNFYY-FYIFQIFLSIIQLHSLGIYHGHIKRENVLIQNNMHIFLTDIN 179

ScVps15          AFIKPVYLPEDNPGEFLFYFDTSKRRTCYLAPERFNSKLYQDGKSNNGRLTKEMDIFSLG 227
PfVps15          ILNDYLYFIP--------------------------KIRYEDERKGRLMKLQEDIFNLG 212

ScVps15          CVIAEIFAEGRPIFNLSQLFKYKSNSYDVNREFLMEEMNSTDLRNLVLDMIQLDPSKRLS 287
PfVps15          ILILEILLRDK---DVSYFFLNENCAYKNDDNFYSGRRKQSGLT-FLKKMESIDEDDKND 268

ScVps15          CDELLNKYRGIFFPDYFYTFIYDYFRNLVTMTTSTPISDNTCTNSTLEDNVKLLDETTEK 347
PfVps15          VENYHHDSNDYHHNENYYNMNQHYDY----------HSDNYYKCNRSNYKEEYTNDENKK 318

ScVps15          IYRDFSQICHCLDFPLIKDGGEIGSDPPILESYKIEIEISRFLNTNLYFPQNYHLVLQQF 407
PfVps15          NGEYQKKYVHALSLPSIKK-----------KKKQIKNEEYPYFNK--------------- 352

ScVps15          TKVSEKIKSVKEECALLFISYLSHSIRSIVSTATKLKNLELLAVFAQFVSDENKIDRVVP 467
PfVps15          ---NQNIKMNIDKYKYYNYHYIN--------------NVNYINIYNDIYNNVSPFSSIDY 395

ScVps15          YFVCCFEDSDQDVQALSLLTLIQVLTSVRKLNQLNENIFVDYLLPRLKRLLISNRQNTNY 527
PfVps15          NGENIFYENDSNFFKRQPIKYSNTHNIKEQLN----DDFQENIKYNDKNIYRQKQNEQVT 451

ScVps15          LRIVFANCLSDLAIIINRFQEFTFAQHCNDNSMDNNTEIMESSTKYSAKLIQSVEDLTVS 587
PfVps15          QKKSQGINIYNVTDLFDDNKDDKYNTGGGDENDNHGNKNDNRGNKNDNDGEKNYNDGEKN 511

ScVps15          FLTDNDTYVKMALLQNILPLCKFFGRERTN----DIILSHLITYLNDKDPALRVSLIQTI 643
PfVps15          YNDGEKNYNDGEKNYNDGEKNYNDGEKNYNDGDKNNISNNADIYYNNLHNSNYYNNVYTK 571

ScVps15          SGISILLGTVTLEQYILPLLIQTITDSEELVVISVLQSLKSLFKTGLIRKKYYIDISKTT 703
PfVps15          KTPNKIKKNDHKDPHMVPYKIWKVVN--QVKNPLIIYSLINHFFSYKNNNKNILKIFNYW 629

ScVps15          SPLLLHPNNWIRQFTLMIIIEIINKLSKAEVYCILYPIIRPFFEFDVEFNFKSMISCCKQ 763
PfVps15          S-YYIFPSTYKYIFFPLSILQLHPLFKNSDFFILLLHFNLPFILFHLDLFSN-------- 680

ScVps15          PVSRSVYNLLCSWSVRASKSLFWKKIITNHVDSFGNNRIEFITKNYSSKNYGFNKRDTKS 823
PfVps15          -KEQDKYILLNMLNRRNNKSGYVSTGKG-SDPAYEKSDNDDDDDDDNNNYNNYNNNNNN 738

ScVps15          SSSLKGIKTSSTVYSHDNKEIPLTAEDINWIDKFHIIGLTEKDIWKIVALRGYVIRTARV 883
PfVps15          NNNNYYYNYHYDDYGNNVRNNHHIGTYLGRLNYYFNIVRTHTTSINKHQMKAYIINTNIN 798

ScVps15          MAANPDFPYNNSNYRPLVQNSPPNLNLTNIMPRNIFFDVEFAEESTSEGQDSNLENQQIY 943
PfVps15          NQIKKQILKQWHSYKKEKKQEYAQYVVNNKN--NVHHNIYIHKKTINNNNNYNNNNNYNN 856

ScVps15          KYDESEKDSNKLNINGSKQLSTVMD-INGSLIFKNKSIATTTSNLKNVFVQLEPTSYHMH 1002
PfVps15          KRSYHSLSYFPFPILSYKNVHAFYKNFLNFYQFLYYSIFYEHKSYMDVFKCMEYYKREIY 916

ScVps15          SPNHGLKDNANVKPERKVVVSNSYEGDVESIEKFLSTFKILPPLRDYKEFGPIQEIVRSP 1062
PfVps15          SPLFNDFYYKQDRKKKETSMMNNMPMKRDLGNEIFACANNIQSNKIHLKKKGKKETCNYI 976

ScVps15          NMGNLRGKLIATLMENEPNSITSSAVSPGETPYLITGSDQGVIKIWNLKEIIVGEVYSSS 1122
PfVps15          NNNNKE------RNNNEHNNNNNPRANENVVRWQFN------EDICQLINIIICCYN--S 1022

ScVps15          LTYDCSSTVTQITMIPNFDAFAVSSKDGQIIVLKVNHYQQESE-VKFLNCECIRKINLKN 1181
PfVps15          LTYNFVKMLTIEIIYCIILHISENRLNNELISFLLYCFNKENEKIKIMVIKCFYKIINNS 1082

ScVps15          FGKNEYAVRMRAFVNEEKSLLVALTNLSRVIIFDIRTLERLQIIENSPRHGAVSSICIDE 1241
PfVps15          MNNR---PNMQLYIEKFLPKYYLLKNQD---IYEKYYYSKYLPLFSYITIQYIYNESLLK 1136

ScVps15          ECCVLILGTTRGIIDIWDIRFNVLIRSWSFGDHAPITHVEVCQFYGKNSVIVVGGSSKTF 1301
PfVps15          NNITKQKKNTLSQIPYMEILNDIKTEIQFILNTQQNYYENLFEFYN-HIFPFCKIMNKKW 1195

ScVps15          LTIWNFVKGHCQYAFINSDEQPSMEHFLPIEKGLEELNFCGIRSLNALSTISVSNDKILL 1361
PfVps15          VKIY-------ILPYLLTNIYKSKNNFIKASCAKVTLKIVFYINQKKVYHIFLQYLNKLL 1248
```

```
ScVps15         TDEATSSIVMFSLNELSSSKAVISPSRFSDVFIPTQVTANLTMLLRKMKRTSTHSVDDSL 1421
PfVps15         FSQNEQ-IIEFLLCELN----HILKKTWQKYKQEQKKSKKFWIFLKKIQLDHLY-IHPSI 1302

ScVps15         YHHDIINSISTCEVDETPLLVACDNSGLIGIFQ 1454
PfVps15         IIQQLVDKIKN-KLKNIEYRTTNKNNKMIFM—1332
```

Figure S3. Sequence alignment of Vps15 proteins. The yeast (ScVps15) and *P. falciparum* (PfVps15) Vps15 protein sequences were aligned using the Clustal W2 program. The conserved amino acid residues are shown in red, the predicted kinase domain of yeast Vps15 is shown in grey shaded bold letters, and the catalytic aspartate residues are highlighted. Notably, PfVps15 has His in place of the first catalytic aspartate residue.
