# Supplementary material for: Characterization of the Autophagy Marker Protein Atg8 Reveals Atypical Features of Autophagy in Plasmodium falciparum
Source: PLoS One. 2014 Nov 26;9(11):e113220. doi: 10.1371/journal.pone.0113220 (PMC4245143; doi:10.1371/journal.pone.0113220)
Supplement: Figure S4 — Alignment of the Atg18 proteins. (PDF) [file pone.0113220.s004.pdf]

```
PfAtg18         MVSLRLDNNRYISFNQDYGCLCMANEKGFKIYNTNPFTQTYSRDLTDRNKNGLYLAEMLY 60
ScAtg18         MSDSSPTIN-FINFNQTGTCISLGTSKGFKIFNCEPFGKFYSEDSG-----GYAIVEMLF 54

PfAtg18         RCNILAITGNKNDKKGKWAKNVLIIWDDRQMREIAKLTFSSNIIGVRLLREIIVVILEYK 120
ScAtg18         STSLLALVG-IGDQPALSPR-RLRIINTKKHSIICEVTFPTSILSVKMNKSRLVVLLQEQ 112

PfAtg18         LCIYRLKDIILLETLNTSKNVSGLCCLSNIDKNIIIAYLSPIKGRVNIHIFEINSSENIH 180
ScAtg18         IYIYDINTMRLLHTIETNPNPRGLMAMSPSVANSYLVYPSPPK--------VINSEIKAH 164

PfAtg18         EELPYIN----------FKT---------------------------------------- 190
ScAtg18         ATTNNITLSVGGNTETSFKRDQQDAGHSDISDLDQYSSFTKRDDADPTSSNGGNSSIIKN 224

PfAtg18         ------NLS-------IYAHDNSIGCINLSNDGKLLVTSSTKGTIIRLFNTFDGTLLNEF 237
ScAtg18         GDVIVFNLETLQPTMVIEAHKGEIAAMAISFDGTLMATASDKGTIIRVFDIETGDKIYQF 284

PfAtg18         RRGTKNAKILSLNISEDNNWLCLTSSRNTVHVFSIYKKKRPLRKVDIICK---------- 287
ScAtg18         RRGTYATRIYSISFSEDSQYLAVTGSSKTVHIFKLG-HSMSNNKLDSDDSNMEEAAADDS 343

PfAtg18         -GKNVSPPALLNYE---------------------------KESKNKKSSLKCLLPCHPY 319
ScAtg18         SLDTTSIDALSDEENPTRLAREPYVDASRKTMGRMIRYSSQKLSRRAARTLGQIFPIKVT 403

PfAtg18         LNSEWS--FASYKLPGKK------ISSICA--------------------------FVND 345
ScAtg18         SLLESSRHFASLKLPVETNSHVMTISSIGSPIDIDTSEYPELFETGNSASTESYHEPVMK 463

PfAtg18         QNCIIVICSNGIIYKLRFNEHIGGDMFKISSHSFD-- 380
ScAtg18         MVPIRVVSSDGYLYNFVMDPERGGDCLILSQYSILMD 500
```

Figure S4. Alignment of the yeast Atg18 (ScAtg18) and its putative *P. falciparum* homolog (PfAtg18) was performed using the Clustal W2 program. The conserved residues are in red font, WD40 repeat domains are shaded, Atg8-family interacting motifs (AIM) are underlined. Both the proteins contain the FRRGT motif (boxed) that has been shown to be essential for binding of ScAtg18 to PtdIns3P and PtdIns(3,5)P2.
