# Supplementary material for: Characterization of the Autophagy Marker Protein Atg8 Reveals Atypical Features of Autophagy in Plasmodium falciparum
Source: PLoS One. 2014 Nov 26;9(11):e113220. doi: 10.1371/journal.pone.0113220 (PMC4245143; doi:10.1371/journal.pone.0113220)
Supplement: Figure S5 — Alignment of Atg8 proteins. (PDF) [file pone.0113220.s005.pdf]

```
PfAtg8          MPS---LKDEVSFENRVAETHKIRSKYPNRIPVVCERAN-RSNLPIIEKKKFLVPMNMLV 56
ScAtg8          MKST--FKSEYPFEKRKAESERIADRFKNRIPVICEKAE-KSDIPEIDKRKYLVPADLTV 57
HLC3B           MPSEKTFKQRRTFEQRVEDVRLIREQHPTKIPVIIERYKGEKQLPVLDKTKFLVPDHVNM 60

PfAtg8          GEFKFILHQHINQSAYGSNMKLFRERTIYLFVN-NIVPKTGLLMQDLYEMYKDEDGYLYM 115
ScAtg8          GQFVYVIRKRIMLPP---------EKAIFIFVN-DTLPPTAALMSAIYQEHKDKDGFLYV 107
HLC3B           SELIKIIRRRLQLNAN---------QAFFLLVNGHSMVSVSTPISEVYESEKDEDGFLYM 111

PfAtg8          EYSCESCLG----- 124
ScAtg8          TYSGENTFGR---- 117
HLC3B           VYASQETFGMKLSV 125
```

Figure S5. Alignment of Atg8 proteins. The *P. falciparum* (PfAtg8), yeast (ScAtg8), and human light chain 3B (HLC3B) proteins were aligned using the Clustal W2 program. Conserved residues are shown in red. ScAtg8 and LC3B are cleaved immediately after the C-terminus Gly residue (highlighted) by Atg4, and the exposed Gly residue is conjugated to PE. This Gly residue is positionally conserved in PfAtg8, and is also the last C-terminus residue, thereby bypassing the processing step.
