# Supplementary material for: Characterization of the Autophagy Marker Protein Atg8 Reveals Atypical Features of Autophagy in Plasmodium falciparum
Source: PLoS One. 2014 Nov 26;9(11):e113220. doi: 10.1371/journal.pone.0113220 (PMC4245143; doi:10.1371/journal.pone.0113220)
Supplement: Figure S6 — Alignment of Atg4 proteins. (PDF) [file pone.0113220.s006.pdf]

```
ScAtg4   MQRWLQLWKMDLVQKVSHGVFEGSSEEPAALMNHDYIVLGEVYPERDEESGAEQCEQDCR    60
PfAtg4   MSKEMKNFEDNQKKIEINSTIQEGKEEENKTLDEIKNVPVAINSNKINKKDNKYIKKKNS    60

ScAtg4   YRGEAVSDGFLSSLFGREISSYTKEFLLDVQSRVNFTYRTRFVPIARAPDGPSPLSLNLL
PfAtg4   KKTKNNFIHIKNKNVDDLYSYQNKSKYKILNYFNNFKYNFSIKRYFKILVNVSLLNYLPL   120

ScAtg4   VRTNPISTIEDYIANPDCFNTDI-------------------------------------    83
PfAtg4   NLRNISNKVYIFGLCLNLKNPDEmkiflilckskilftyrsnflirindnnlyrsnniml   180

PfAtg4   vdnnvsqsniftydnttynnsnitinnnnnnnsnitinnnnnnnnnssnitinnnisnlc
         innnnhtstsnttnssdnslpilcqeyehidqqdyliyiskkkkkkkklkkkkiyykqri
         inftdipehflkkiyfddkecfyinfkksskghndtytyqkqhsqeikenyslqnnkkkk
         skeeiygdtctyttsesnktiiylenskkcnsvdnkeiknkniskddmhyyreqkkkiik
         nksenntcdddlecfnnpdndrndpneemkkkkkkikinnnnnnieseatikniniiktk
         knslkilhsrskkhsvlkikkknknniiknnninikeylkyvnqnitksqiyekdkkyic   540

ScAtg4   ----GWGCMIRTGQSLLGNALQILHLGRDFRVNGNESLERESKFVNWFNDTPEAPFSLHN   199
PfAtg4   MSDNGWGCMIRVIQMVLANILIHFNISNRYVYFHNVNDYILYKNYINKLTTCTNKENKII   600

ScAtg4   FVSAGTELSDKRPG----------------------------------------------   213
PfAtg4   QIEEKTMNKDKEESiylnyknkdstkemvkheknnqcesnecnlnknasqnnkesdtlmi   660

PfAtg4   hpqsniingnnnninsnnnnnnnnnnsnnnnnsnnnnnnssssttfsnsntylvpdsfkq
         eynkssiktdflqsdkqitlndtninesqgsdiynidnvenitnkynssdlhhcenynnf
         llnnknnnndsnseimhinnsliysilsefrdleqakysiqniiyemikykkiddnqieh   840

ScAtg4   ---EWFGPAATARSIQSLIYGFPECGIDDCI-----------------------------   241
PfAtg4   FVHDWLGPTSSAIIISNLINKKKVRFVKKNKmknnfrgtnihmdkniyiekeqkeltnyt   900

ScAtg4   --------------------------------------------------VSVSSGDIYE   251
PfAtg4   nnnkqiykllnqkeniniksddnikndkkkynkllflkekkkkytffsVAFETGVIYN   960

ScAtg4   NEVEKVFAENPNSRILFLLGVKLGINAVNESYRESICGI–LSSTQSVGIAGGRPSSSLY    309
PfAtg4   NKVLKFFQIKQDIFIIIWICLKLGIDSLNVSKYKKSLLSCFLLKQFQGISSGNTNTSAHY  1020

ScAtg4   FFGYQGNEFLHFDPHIPQPAVEDSFVESCHTSKFGKLQLSEMDPSMLIGILIKGEKDWQQ   369
PfAtg4   FYSANDNGLFYLDPHIKCQNAFIDFNENISSQFFMHKVKFLPWEY---------------  1065

ScAtg4   WKLEVAESAIINVLAKRMDDFDVSCSMDDVESVSSNSMKKDASNNENLGVLEGDYVDIGA   429
PfAtg4   -------------LNSSLSLIFVVQSKDDYFNLIQNLKLIDSSLFEIYHEEPQYVYKNEL

ScAtg4   IFPHTTNTEDVDEYDCFQDIHCKKQKIVVMGNTHTVNANLTDYEVEGVLVEKETVGIHSP   489
PfAtg4   NYDTDDSGLVVL------------------------------------------------  1124

ScAtg4   IDEKC                                                          494
PfAtg4   -----
```

Figure S6. Alignment of Atg4 proteins. The yeast (ScAtg4) and *P. falciparum* (PfAtg4) sequences were aligned using the Clustal W2 program. Shown are the conserved residues (red), and catalytic cysteine and histidine residues (highlighted). The inserts in PfAtg4 are shown in lower case.
