# Supplementary material for: Characterization of the Autophagy Marker Protein Atg8 Reveals Atypical Features of Autophagy in Plasmodium falciparum
Source: PLoS One. 2014 Nov 26;9(11):e113220. doi: 10.1371/journal.pone.0113220 (PMC4245143; doi:10.1371/journal.pone.0113220)
Supplement: Figure S7 — Alignment of Atg7 proteins. (PDF) [file pone.0113220.s007.pdf]

```
PfAtg7          MKKKFEEENKPSYILKHNNNEFKIDISYFTQLHEHKINIYKLQSNYVNLCSSTYVNKIKL 60
ScAtg7          ----MSSERVLSYAPAFKS---FLDTSFFQELSRLKLDVLKLDSTCQPLTVNLDLHNIPK 53

PfAtg7          GFKYKLLNRYLIEFAHPFIHVRTIEINKKSFLKYENFDNEDEKNNMEPNDCTKTIENERN 120
ScAtg7          SADQVPL-----------------------FLTNRSFEKHNNKRTNEVP----------- 79

PfAtg7          HINNINDGNKKVQKIWYIMNNYRNNYLGVLLNFNTLEEFLKCNKDDHINYTLEPLKCYIN 180
ScAtg7          -------------------------LQGSIFNFNVLDEFKNLDK--QLFLHQRALECWED 112

PfAtg7          NEKNDICKDMNLYIHDNIYDDTFWEYKENCLTVLEKINKYVILSFFDLKKYICYYSIANP 240
ScAtg7          G----------------------------IKDINKCVSFVIISFADLKKYRFYYWLGVP 143

PfAtg7          IIKPKDNYYKLIKNSTRYFFYIDSKYVYINTENRHINIIDIFYLSYKIDDYFNNYKMFLN 300
ScAtg7          CFQRP------------------------------------------------------S 149

PfAtg7          TNIFLLLKFDNIPLHTMNNQDYYDEYINKLYTNIECEEDQKSKKEFYQINSFYKLFEYLK 360
ScAtg7          STVLHVRPEPSLKGLFSKCQKWFDVNYSKWVCILDADDEIVN------------------ 191

PfAtg7          LNDISQNSYHPMGNKSFNNHYNNNSSMLHKNYDMVILPINALSELKEDIKNSKDKILRYI 420
ScAtg7          ----------------------YDKCIIRKTKVLAIRDTSTMENVPSALTKNFLSVLQYD 229

PfAtg7          KKDFFDLYICFIDINYIFNSLSWDFRNLLYCLTLKYKLYDFQIDVLAFRDISLLRQQYVG 480
ScAtg7          VPDLIDFKLLIIRQ---------------------------------------------- 243

PfAtg7          TFKSQEGFIWSYPKVVMKRGSINNPRNYNDEDKNNDNNNYDDKNNDNNNYDDSHNNNYDD 540
ScAtg7          ------------------------------------------------------------

PfAtg7          KNNDNNNYDDSHNNNYEDSHNNNYDDSDLHKDIDMDKDKNNSFHYNPINNCLSHQDVSFC 600
ScAtg7          ------------------------------------------------------------

PfAtg7          SVTKMCKVNYNSIKDCKNDWRDDLTNEYSHDMNPIHEDIEHSSSQYENNMSVNNTYKKDN 660
ScAtg7          -------------------------NEGSFALNATFASIDPQSSSSNPDMKVS------- 271

PfAtg7          RNIKHNHNNIYHNHLVKYILNSSLFQVTVPDKVHFIYDNGSNYVDINLNGKKDDSLNKQD 720
ScAtg7          ------------------------------------------------------------

PfAtg7          IHILEKKKEGDTCIINSYLKSFSDEKKNDCIDVSSNLGFSINIRKEDNHFTTRVKYKDEE 780
ScAtg7          ------------------------------------------------------------

PfAtg7          MDVLHISEGDENENNMNNATNNNINNNIIKNYKTFCCDNKVYDILCGWKYYEDKKKEKKS 840
ScAtg7          ----------------------------------------------GWERNVQGKLAPR- 284

PfAtg7          IISIINLNDFINKDTIQRISLELNIKLIKWKILKDLKFEHIKKLKILIIGLGTLGCMVAR 900
ScAtg7          ---VVDLSSLLDPLKIADQSVDLNLKLMKWRILPDLNLDIIKNTKVLLLGAGTLGCYVSR 341

PfAtg7          NCVSWGIQHYTFVDNSRVSFSNISRQYLYTLEDAEKYGNIGEYKCVAAKKNLLKICPDLN 960
ScAtg7          ALIAWGVRKITFVDNGTVSYSNPVRQALYNFEDCGKP------KAELAAASLKRIFPLMD 395

PfAtg7          ITAKVMDIPMPGHLNYLNENLEDTINELDNLINNHDVVFLLTDSKESRYFPCLMIAEKQY 1020
ScAtg7          ATGVKLSIPMIGHKLVNEEAQHKDFDRLRALIKEHDIIFLLVDSRESRWLPSLLSNIENK 455

PfAtg7          NSLKELQESVNHNNNNNNNNNSSSSSSGSNKFRKGDNVLCEEENMITHEYIENIKCTKIM 1080
ScAtg7          T----------------------------------------------------------- 456

PfAtg7          DKSLNNILLYEQNNNIYKSLNNIHMYDRYQEIFYNNILTSVKRLCKMPPLGITVAISFDS 1140
ScAtg7          --------------------------------------------------VINAALGFDS 466

PfAtg7          FVVLRHSYLYFKG----ACYFCNDMHCPSDSLSYRTLDEKCTVTRCGISNISSSIATELL 1196
ScAtg7          YLVMRHGNRDEQSSKQLGCYFCHDVVAPTDSLTDRTLDQMCTVTRPGVAMMASSLAVELM 526

PfAtg7          LALTQHPLYFFAPHIDRDQYIYNYDNDMNQKKNSDISNIFTSCLGATPHIMNFNLANFTI 1256
ScAtg7          TSLLQT---------------------------KYSGSETTVLGDIPHQIRGFLHNFSI 558

PfAtg7          KKIFCEPFEKCMCCSERVILKYQEDKMDFIRNVIRDSSILERITNMDQLKVEEN----DV 1312
ScAtg7          LKLETPAYEHCPACSPKVIEAFTDLGWEFVKKALEHPLYLEEISGLSVIKQEVERLGNDV 618

PfAtg7          IILE-------- 1316
ScAtg7          FEWEDDESDEIA 630
```

**Figure S7. Alignment of Atg7 proteins.** The sequences of yeast (ScAtg7) and *P. falciparum* (PfAtg7) were aligned using the Clustal W2 program. Conserved residues are shown in red, the ATP-binding pockets are underlined, and the catalytic cysteine residues are highlighted with black background.
