# Supplementary material for: Characterization of the Autophagy Marker Protein Atg8 Reveals Atypical Features of Autophagy in Plasmodium falciparum
Source: PLoS One. 2014 Nov 26;9(11):e113220. doi: 10.1371/journal.pone.0113220 (PMC4245143; doi:10.1371/journal.pone.0113220)
Supplement: Figure S8 — Sequence alignment of Atg3 proteins. (PDF) [file pone.0113220.s008.pdf]

```
PfAtg3          MSDQINVKHKIGDTCRKLYSYFKTVNNTSTFIQNGTLTPSEFVDSGDFLVYKFKTWEWQE 60
ScAtg3          ---------MIRSTLSSWREYLTPITHKSTFLTTGQITPEEFVQAGDYLCHMFPTWKWNE 51

PfAtg3          ADKDR-VVPYLPENKQFLITKNVPCKQRIKDLNNIVHDLKIVDNDWLLPSYEEDNNPTDI 119
ScAtg3          ESSDISYRDFLPKNKQFLIIRKVPCDKRAEQC------VEVEGPDVIMKGFAEDGDEDDV 105

PfAtg3          YEYLPNSEYTINDKNVQNVQNIYNYEEEEEDDNCDEAIDINNFYMENNLIKEHDPASINS 179
ScAtg3          LEYIGSETEHVQSTPAGGTK--------------DSSIDDIDELIQDMEIKEED-ENDDT 150

PfAtg3          TSCYSKNMLHDNLMKIRTYDVSITYDKYYQTPRIWLFGYNENGDPLKSEEIFEDILSDYS 239
ScAtg3          EEFNAKGGLAKDMAQERYYDLYIAYSTSYRVPKMYIVGFNSNGSPLSPEQMFEDISADYR 210

PfAtg3          YKTVTYDPHPCTG--VMTASIHPCKHA-----------------------EAILNVVNNW 274
ScAtg3          TKTATIEKLPFYKNSVLSVSIHPCKHANVMKILLDKVRVVRQRRRKELQEEQELDGVGDW 270

PfAtg3          ISEEKEP----RHDLYLLFLLKFISGVIPTIEYDFTTDIEIPRDSNAGL 319
ScAtg3          EDLQDDIDDSLRVDQYLIVFLKFITSVTPSIQHDYTMEGW--------- 310
```

Figure S8. Sequence alignment of Atg3 proteins. The sequences of yeast (ScAtg3) and *P. falciparum* (PfAtg3) were aligned using the Clustal W2 program. Conserved residues are shown in red, the predicted catalytic cysteine residues are highlighted, and the Atg8 family interacting motifs (AIM) are underlined.
