# Supplementary material for: Characterization of the Autophagy Marker Protein Atg8 Reveals Atypical Features of Autophagy in Plasmodium falciparum
Source: PLoS One. 2014 Nov 26;9(11):e113220. doi: 10.1371/journal.pone.0113220 (PMC4245143; doi:10.1371/journal.pone.0113220)
Supplement: Figure S9 — Sequence alignment of Atg12 proteins. (PDF) [file pone.0113220.s009.pdf]

```
ScAtg12         MSRILESENETESDESSIISTNNGTAMERSRNNQELRSSPHTVQNRLELFSRRLSQLGLA 60
PfAtg12         ----------------------------------------MTENYIQYIP--------- 10

ScAtg12         SDISVDQQVEDSSSGTYEQEETIKTNAQTSKQKSHKDEKNIQKIQIKFQPIGSIGQLKPS 120
PfAtg12         ---------------VFEKKPDMREILINRRN---------QKIKIVFKCISGTTILKKN 46

ScAtg12         VCKISMSQSFAMVILFLKRRL-KMDHVYCYINNSFAPSPQQNIGELWMQFKTNDELIVSY 179
PfAtg12         KVLINGNETFSSLLIFLKRIFNKNDNIYLYINNNIKPNLDDYIYDLYDLYQISGSLNISY 106

ScAtg12         CASVAFG 186
PfAtg12         SFTPAY- 112
```

Figure S9. Sequence alignment of Atg12 proteins. The yeast (ScAtg12) and *P. falciparum* (PfAtg12) sequences were aligned using the Clustal W2 program. Conserved residues are shown in red. The C-terminus Gly residue of ScAtg12 (absent in PfAtg8), which is conjugated to Atg5 protein, is highlighted.
