# Supplementary material for: Characterization of the Autophagy Marker Protein Atg8 Reveals Atypical Features of Autophagy in Plasmodium falciparum
Source: PLoS One. 2014 Nov 26;9(11):e113220. doi: 10.1371/journal.pone.0113220 (PMC4245143; doi:10.1371/journal.pone.0113220)
Supplement: Figure S10 — Sequence alignment of Atg5 proteins. (PDF) [file pone.0113220.s010.pdf]

```
PfAtg5          MEIGYMEVPNIKDINSNIEKSGLVLCVSLNQKESESLISPSYYYIYVHRYMYLSNIIPKC 60
hAtg5           -------MTDDKDVLRDVWFGRIPTCFTLYQDEITEREAEP-YYLLLPRVSYLTLVTDKV 52
ScAtg5          -------MNDIKQLLWNGELN-VLVSIDPSFLMKGSPREIAVLRIRVPRETYLVNYMPLI 52

PfAtg5          LEFFKSFILPFYGNKFGVYFECIKKEQKKSSNNNNNNNNNITYTSTNNYEEKIVLDWRLP 120
hAtg5           KKHFQKVMR----------------------QEDISEIWFEYEGTP-------LKWHYP 82
ScAtg5          WNKIKSFLS------------------FDPLTDSEKYFWFEHNKTP-------IPWNYP 86

PfAtg5          IGVLFDIYCDLDKSQKEYIKTYEKFNDTNINMNNKKNITICSNNELFLNHINIVKLKGTD 180
hAtg5           IGLLFDLLASS------------------------------------------------- 94
ScAtg5          VGVLFDCLAGKS------------------------------------------------ 98

PfAtg5          KNYDKKSNERQNQKNIYDANRNISTNTNNSSNVNHQQNCDRTDDDTLHKGDDILHKGDDI 240
hAtg5           ------------------------------------------------------------
ScAtg5          ------------------------------------------------------------

PfAtg5          LHKGDDILHKGDDILHKGDDILYKGNDILHKGDDILHKGDDILHTDAFINEKETKEGTDK 300
hAtg5           ------------------------------------------------------------
ScAtg5          ------------------------------------------------------------

PfAtg5          KKQINRNIKNEERERGKEVKNIIVENYYEENYMKEKKIIVENGKGSTKNDVEENKKDINT 360
hAtg5           ------------------------------------------------------------
ScAtg5          ------------------------------------------------------------

PfAtg5          HYDDIKLNNVDIYDDIKLNNVDIYDDTADFQYIQFIQNEKINNEWYNKQFVNISNKNIPW 420
hAtg5           --------------------------------------------------------ALPW 98
ScAtg5          -----------------------------------------------------------A 99

PfAtg5          MLIVHFKGEEEYPLSIINKKYDEKNTHFKGDINILPYNNYIPLYKGFNNFEEYIINQLKK 480
hAtg5           NITVHFKS------------FPEK------DLLHCPSK---------DAIEAHFMSCMKE 131
ScAtg5          TFTTSFENQVKDVLTFLRIHLVMGDSLPPTIIPIASSK---------TQAEKFWFHQWKQ 150

PfAtg5          ANCILNKNNRALEILPQRIQKDILYSLKHFHIEKICSLYREYIDYNMLNFINYFNNSYIK 540
hAtg5           ADALKHKS-QVINEMQKKDHKQLWMGLQNDRFDQFWAINRKLMEY--------------- 175
ScAtg5          VCFILNGSSKAIMSLSVNEARKFWGSVITRNFQDFIEISNKISSS--------------- 195

PfAtg5          KVQQVCDLNRHKDNSIQINGKSSGKQNEENLCLLKMDLHEEESPVKITHCKDILNNDNEN 600
hAtg5           --------------------------------------PAEENGFR-------------- 183
ScAtg5          --------------------------------------RPRHIPLII------------- 204

PfAtg5          YDNENNNNENNNEHNCHRNSYDVHLNENFINDQSQKNHISNIRTSYNNDHVSDSLQTDIQ 660
hAtg5           ------------------------------------------------------------
ScAtg5          ------------------------------------------------------------

PfAtg5          NDFSSIILENTKEQGDHINFKNNLHNNEKYPNVYDILKDEKVLKDCPIILHIYGPPYNQI 720
hAtg5           ----------------YIPFR------------------------------IY-----QT 192
ScAtg5          ----------------------------------------------------------QT 206

PfAtg5          LTKYPFLKLIHSNNNNNNNNNDNKDNNNNSNKYDGCKKYIQNVHLNTLGDFLHEQLPSFV 780
hAtg5           TTERPFIQKLFRPVAADG-------------------------QLHTLGDLLKEVCP--- 224
ScAtg5          SRTSGTFRISQPTISMTG-----------------------------VNPTLKDIEG--- 234

PfAtg5          RKIINKDEKNETSHILNKYDKDKYLNSETIYYFIEDDYLIFSPYMFIIVNGIQIPLNTPL 840
hAtg5           -SAIDPEDGEKKNQ-------------------------------VMIHGIEPMLETPL 251
ScAtg5          -DILDVKEGINGND------------------------------VMVICQGIEIPWHMLL 263

PfAtg5          YWLAANFSQFDHFLHITIRIPPY-------- 863
hAtg5           QWLSEHLSYPDNFLHISIIPQPTD------- 275
ScAtg5          YDLYSKLRSFDGFLYITLVPIKGGDKASSEL 294
```

Figure S10. Sequence alignment of Atg5 proteins. The sequences of yeast (ScAtg5), human (hAtg5), and *P. falciparum* (PfAtg5) were aligned using the Clustal W2 program. Conserved residues are shown in red. The K^{130} of hAtg5 that is conjugated to the extreme C-terminus G of Atg12 is highlighted, and this residue is positionally conserved in all three proteins.
