# Supplementary material for: Characterization of the Autophagy Marker Protein Atg8 Reveals Atypical Features of Autophagy in Plasmodium falciparum
Source: PLoS One. 2014 Nov 26;9(11):e113220. doi: 10.1371/journal.pone.0113220 (PMC4245143; doi:10.1371/journal.pone.0113220)
Supplement: Figure S11 — Alignment of the Atg23 proteins. (PDF) [file pone.0113220.s011.pdf]

```
ScAtg23         ------------------------------------------------------------
PfAtg23         MTKSICELQNKNSISTELLQEIKKMENELKRKNDEYEKLNANYREIYGSYILAKTNLDDT 60

ScAtg23         --------------------------MELNQVLEKKEQILQYLG--------------TL 20
PfAtg23         TNNYEGEILKLETMLKEKEHEYMDLRINFQELLEEKCKLENDMDNYEKAIYEINENLISL 120

ScAtg23         -VGLHEKALSDVNSA-----SQVTSIRKDITICLN---DLCRIN---------------- 55
PfAtg23         KEAHHKEELKEVHEENTNLYSTIDKFKEEVEYMKENYKQLEKMNQDKINTIENLEKEMMDM 180

ScAtg23         -----------DLLVSHDGLLKREIGSLLRDKQELLELNE------------REQLLWKE 92
PfAtg23         KEEKKMVGEKLDLVQSQQRMIQMEIKNLNKEKEQLKEKEETLKNEKEKVKQCKEVLKIEE 240

ScAtg23         RKSWHIKQETDAAPADYVIDKDAIITISS---HHRTSLNKYIESVGAENTILSNTDD--- 146
PfAtg23         KQIDTNKQVLDKEKEQIEKNKEELKLMQQNLNKEKEQIEKNKQELDKEKQIYENDKKNIM 300

ScAtg23         ---------SDAMIEEVQNAESSADQMIRNYKLLQLSHKQAKSEIIRLETLLRDFK---- 193
PfAtg23         LKNEDLKKLLDEYKEEIMNKEGHIKQIEEKILLLEDEKKNFQNKIDILDNKMQEFKNEIT 360

ScAtg23         ---KDNKFIEEELKRQSGRIRSEMGNIDFHLSKIEESKHQLMKRIGFESPLTQEKSLSEK 250
PfAtg23         NEKKLNYQLKDDIDNKQNEVIKRDSIIEILYNKLKEKENDITLFQNENLSLRNCQKELNK 420

ScAtg23         IFNLR----LSSADEDYNERQTINMKNFVHMKD--------------------------- 279
PfAtg23         ILDNKKNEMINIEKDLYEKLAKLLIKKKIRQRDDLHFDGHPQSSILKILWFGYVDICKCI 480

ScAtg23         -------------------------LIELKIEDLQEQLMRNKNESSTVLTQR-------- 306
PfAtg23         LKNRDGYLYKGNKNHRFICAYDTDLFLDINVENTKKGLLSFKNETRGIFQLNDEEAKKLV 540

ScAtg23         --------------------------------------------ELWLDCQKKVGDLESK 322
PfAtg23         YVGPFNKFKGFNFCISSNKNNLEIVKGNKCTYIFEEMYVTKKGTKLVLIKEKESGKYFSG 600

ScAtg23         LITKLRSSSNSK-IPPNEMSEMINSTIQYLNN---------------------------- 353
PfAtg23         LNKNLYLEKKKRGVDKLDYNNVVNKLINYIANEKEENCNMIIKTSDQKLKIENEKNTCLV 660

ScAtg23         -------------------------------LLDSSDEKLTTTLISNERDVLSKACEELH 382
PfAtg23         QKKKNSDNSYNNEEKTNLVTKRSKTPIRKENSLHSKYDKTKTTKITNQLKKYDSKEKQNK 720

ScAtg23         SESTTAQDGSSALPSKPID----------------------------------------- 401
PfAtg23         EKSTSEKQTGKKLASEKNNEEKLTNGKINEDKLNNEKNNEDKLNNNRELSDENLHKYKIA 780

ScAtg23         -----------------------------------IHKSHKGSNASSNLKQPSTPSFLVA 426
PfAtg23         KEMLLLSDSNSSSTCDDENIYHLKNKYNHTEQNEFIHNNVINKSIATNYIENINTSLFKY 840

ScAtg23         SKSPPKIGISESVVNANKNDAISKKVE--------------------------------- 453
PfAtg23         SKCSENYNNTNIVLTTNKEEAILFEIFNYEQIVEHDAIKLASECVFNFLLNYNKSNLHDL 900

ScAtg23         --------------------------
PfAtg23         SNIGSEGNSNIFSGIDDDWCILPAYL 926
```

Figure S11. Alignment of the yeast Atg23 (ScAtg23) and its putative *P. falciparum* homolog (PfAtg23) was performed using the Clustal W2 program. Conserved amino acid residues are shown in red, and the putative Atg8 family interacting motif (AIM) in PfAtg23 is underlined.
