# Supplementary material for: Characterization of the Autophagy Marker Protein Atg8 Reveals Atypical Features of Autophagy in Plasmodium falciparum
Source: PLoS One. 2014 Nov 26;9(11):e113220. doi: 10.1371/journal.pone.0113220 (PMC4245143; doi:10.1371/journal.pone.0113220)
Supplement: Figure S12 — Expression of recombinant PfAtg8. (PDF) [file pone.0113220.s012.pdf]

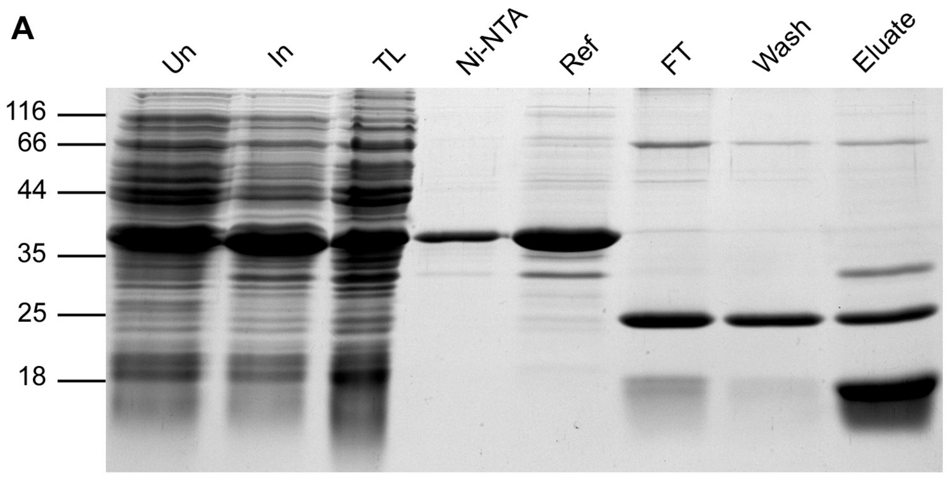
A
Un
In
TL
Ni-NTA
Ref
FT
Wash
Eluate
116
66
44
35
25
18

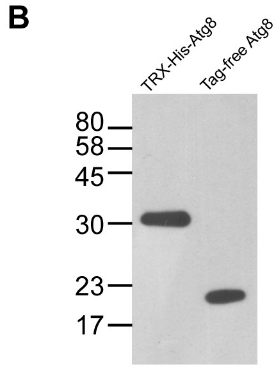
B
TRX-His-Atg8
Tag-free Atg8
80
58
45
30
23
17

Figure S12

Figure S12. Expression of recombinant PfAtg8. PfAtg8 was expressed in *E. coli*, and antibodies were generated against the recombinant protein. **A.** The entire PfAtg8 coding sequence was expressed as a thioredoxin-His (Trx-His-Atg8) fusion protein in BL21(DE3) cells, purified under denaturing conditions, and refolded. The refolded protein was digested with thrombin, to remove the Trx-His fusion-tag. The flow through and wash fractions predominantly contained PfAtg8, whereas the eluate was enriched with the fusion tag. The coomassie stained SDS-PAGE shows lysates of uninduced (Un) and induced (In) bacteria, total lysate of the induced bacteria (TL), purified Trx-His-Atg8 fusion protein (Ni-NTA), refolded fusion protein (Ref), PfAtg8 in the flow through (FT) and wash (Wash) fractions of the thrombin digested refolded protein sample, and Trx-His fusion-tag in the eluate fraction. The flow through and wash fractions were pooled and used for generation of antibodies. **B.** Western blot of purified Trx-His-Atg8 (Trx-Atg8) and tag-free (Atg8) recombinant PfAtg8 (2 µg/lane) using anti-Atg8 antibodies indicates reactivity of antibodies with both the proteins. M, molecular weight in kD.
