# Supplementary material for: Characterization of the Autophagy Marker Protein Atg8 Reveals Atypical Features of Autophagy in Plasmodium falciparum
Source: PLoS One. 2014 Nov 26;9(11):e113220. doi: 10.1371/journal.pone.0113220 (PMC4245143; doi:10.1371/journal.pone.0113220)
Supplement: Figure S13 — Expression and localization of Atg8 in sexual erythrocytic stages. (PDF) [file pone.0113220.s013.pdf]

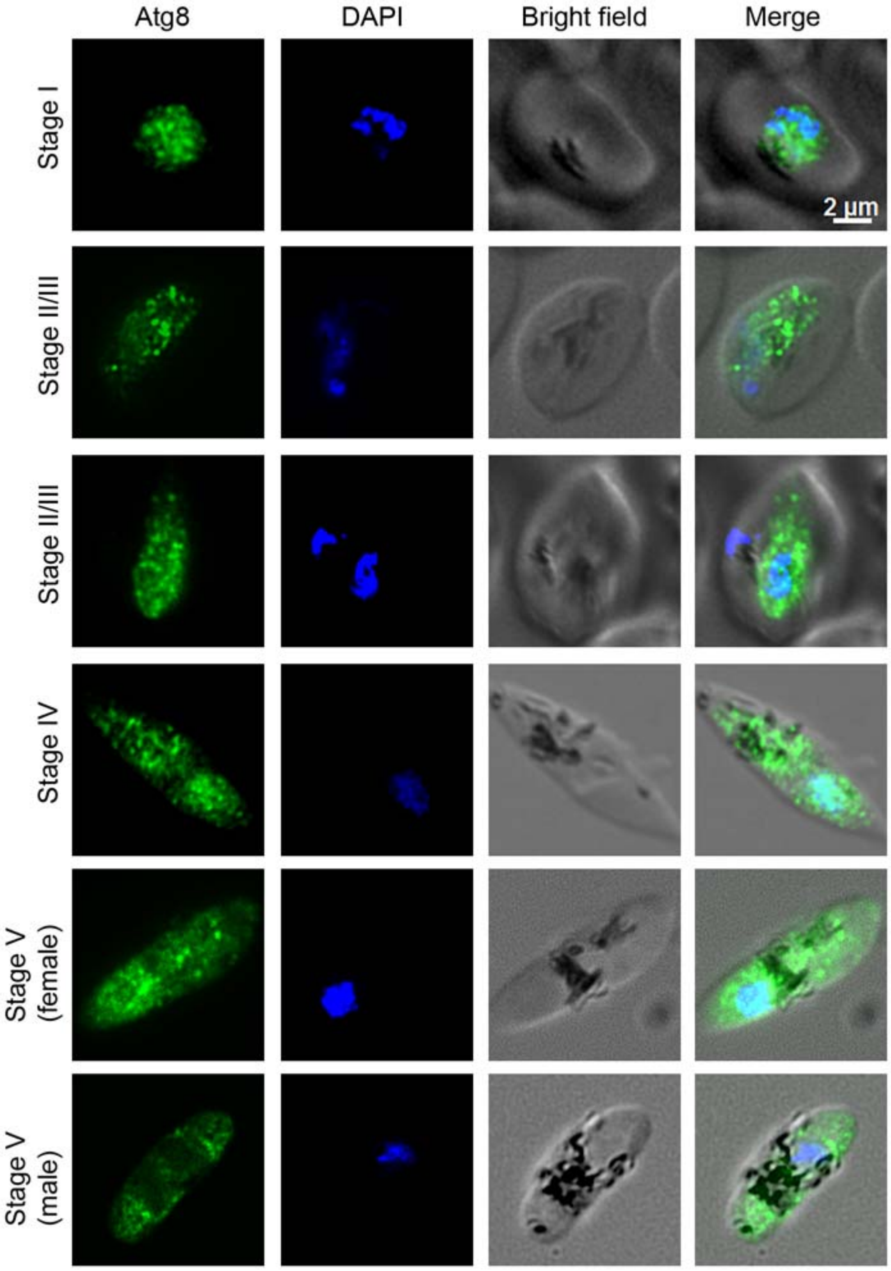
Atg8
DAPI
Bright field
Merge
Stage I
2 µm
Stage II/III
Stage II/III
Stage IV
Stage V (female)
Stage V (male)

Figure S13

Figure S13. Expression and localization of Atg8 in sexual erythrocytic stages. Anti-Atg8 antibodies were used to detect PfAtg8 in the indicated gametocyte stages of *P. falciparum* by IFA as described in Materials and Methods section. The labels for indicated stages are as described in Figure 2, and all images indicate expression of Atg8, which appears to be associated with puncta throughout the parasite.
