# Supplementary material for: Characterization of the Autophagy Marker Protein Atg8 Reveals Atypical Features of Autophagy in Plasmodium falciparum
Source: PLoS One. 2014 Nov 26;9(11):e113220. doi: 10.1371/journal.pone.0113220 (PMC4245143; doi:10.1371/journal.pone.0113220)
Supplement: Figure S14 — Effects of stresses on autophagy. (PDF) [file pone.0113220.s014.pdf]

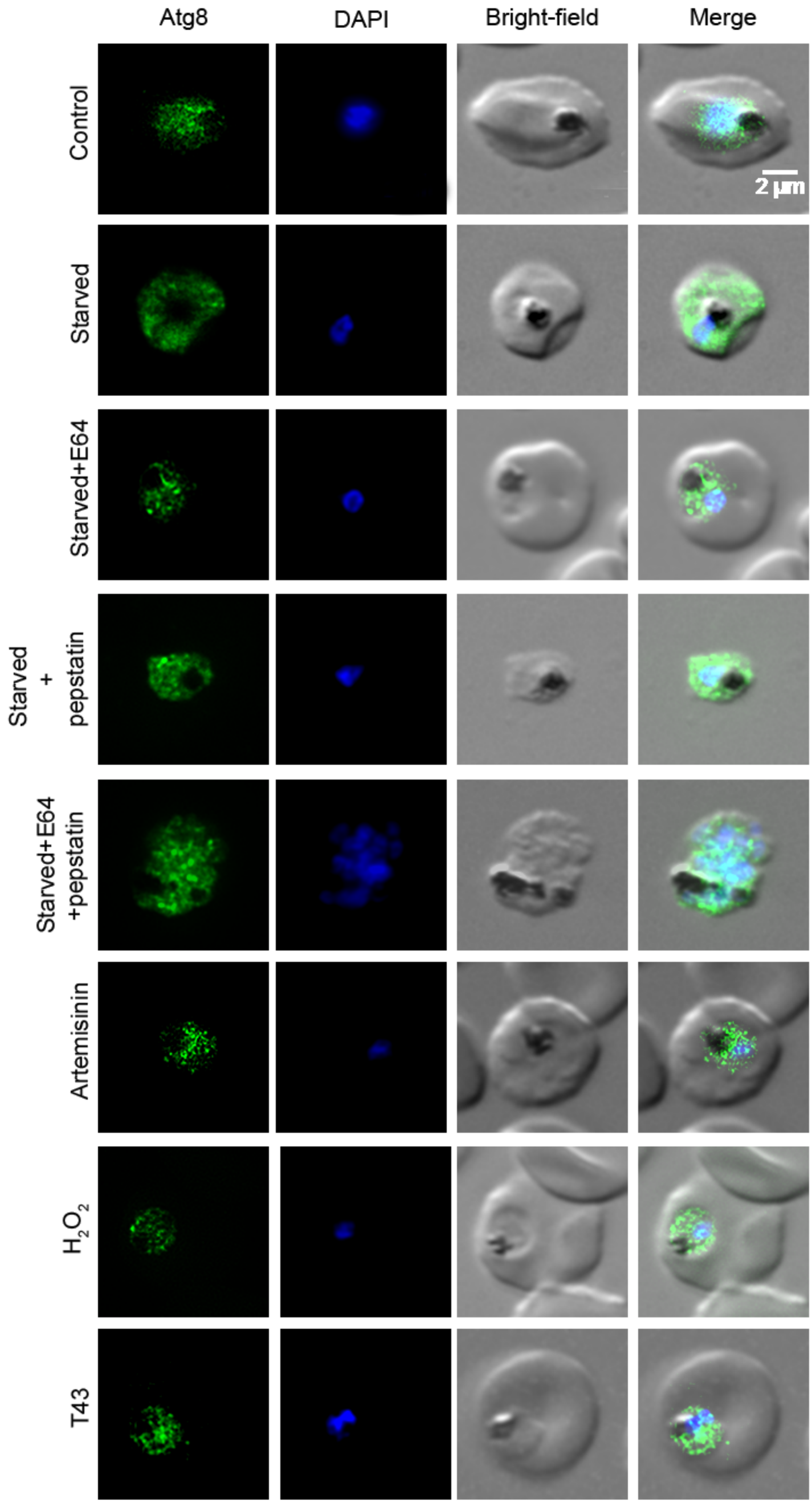

Atg8
DAPI
Bright-field
Merge
Control
2 µm
Starved
Starved+E64
Starved + pepstatin
Starved+E64 +pepstatin
Artemisinin
H2O2
T43

Figure S14

Figure S14. Effects of stresses on autophagy. Early/mid trophozoite stage parasites were exposed to a variety of stresses by culturing in complete medium (Control) or in HBSS containing DMSO (Starved) or the indicated inhibitors (22 µM E64 (Starved + E64), 220 µM pepstatin (Starved + pepstatin), or both (Starved + E64 + Pep) at 37°C for 8 hours. For oxidative stress, parasites were grown in complete medium containing the indicated oxidative stress-causing agents (90 nM artemisinin (Artemisinin), 100 µM H_2O_2 (H_2O_2)) at 37°C for 8 hours. For heat shock (T43), parasites were cultured in complete medium at 43°C for 8 hours. After 8 hours of the indicated treatments, parasites were evaluated for localization of Atg8 by IFA using anti-Atg8 antibodies as described in the Materials and Methods section. The images of indicated treated parasites show the presence of Atg8 specific signal (Atg8), nucleic acid staining (DAPI), the parasite and the erythrocyte boundaries (Bright field), and the merged of all three images (Merge). The Atg8 signal is present throughout the parasite regardless of the treatment, and the signal patterns in control and treated parasites are similar.
