# Supplementary material for: Characterization of the Autophagy Marker Protein Atg8 Reveals Atypical Features of Autophagy in Plasmodium falciparum
Source: PLoS One. 2014 Nov 26;9(11):e113220. doi: 10.1371/journal.pone.0113220 (PMC4245143; doi:10.1371/journal.pone.0113220)
Supplement: Table S1 — Identity of P. falciparum Atg proteins with other Plasmodium homologs. (PDF) [file pone.0113220.s015.pdf]

Table S1. Percentage sequence identity of *P. falciparum* Atg proteins with other *Plasmodium* homologs. Amino acid sequences of putative *P. falciparum* Atg proteins were aligned using the Jalview program. Shown are the sequence identities of *P. falciparum* Atg proteins with homologs in other *Plasmodium* species. The gene ID is in the bracket.

P. falciparum	***P. vivax***	***P. knowlesi***	***P. berghei***	***P. yoelii***	***P. chabaudi***
Atg1 (PF3D7_1450000)	94.4 (PVX_117985)	94.2 (PKH_125630)	92.8 (PBANKA_131370)	92.8 (PYYM_1314500)	92.8 (PCHAS_131700)
Atg3 (PF3D7_0905700.1)	67.9 (PVX_098725)	67.9 (PKH_070290)	67.8 (PBANKA_041570)	66.3 (PYYM_0418500)	68.1 (PCHAS_041660)
Atg4 (PF3D7_1417300)	35.9 (PVX_085585)	36.5 (PKH_133050)	39.6 (PBANKA_102540)	40.2 (PYYM_1027500)	40.2 (PCHAS_102620)
Atg5 (PF3D7_1430400)	29.5 (PVX_085020)	30.1 (PKH_131990)	34.0 (PBANKA_101430)	35.0 (PYYM_1015800)	35.1 (PCHAS_101510)
Atg7 (PF3D7_1126100)	32.6 (PVX_091922)	35.3 (PKH_092370)	44.0 (PBANKA_092220)	42.7 (PYYM_0923600)	41.7 (PCHAS_092220)
Atg8 (PF3D7_1019900)	88.7 (PVX_001860)	88.7 (PKH_060390)	87.9 (PB000658.01.0)	87.9 (PYYM_0504500)	89.5 (PCAS_050420)
Atg11 (PF3D7_0216700.1)	30.1 (PVX_002705)	32.5 (PKH_040340)	36.4 (PBANKA_031340)	36.3 (PYYM_0314200)	34.4 (PCHAS_031550)
Atg12 (PF3D7_1470000)	68.8 (PVX_116967)	69.6 (PKH_123525)	68.8 (PBANKA_133320)	68.8 (PYYM_1334900)	68.8 (PCHAS_133780)
Atg15 (PF3D7_1427100)	37.0 (PVX_085180)	38.1 (PKH_132330)	42.1 (PBANKA_101750)	42.8 (PYYM_1019000)	43.3 (PCHAS_101830)
Atg17 (PF3D7_1120000)	42.0 (PVX_091635)	46.7 (PKH_091740)	45.6 (PBANKA_092820)	50.9 (PYYM_0929600)	50.0 (PCHAS_091610)
Atg18 (PF3D7_1012900)	97.9 (PVX_094865)	97.1 (PKH_081240)	91.6 (PBANKA_121130)	91.6 (PYYM_1213900)	91.1 (PCHAS_121200)
Atg22 (PF3D7_0629500)	60.5 (PVX_114575)	59.3 (PKH_111940)	52.2 (PBANKA_112830)	53.9 (PYYM_1130600)	51.9 (PCHAS_112780)
Atg23 (PF3D7_1126700)	45.0 (PVX_091950)	45.2 (PKH_092430)	45.4 (PBANKA_092170)	46.5 (PYYM_0923100)	46.7 (PCHAS_092270)
VPS34 (PF3D7_0515300)	36.4 (PVX_080480)	36.0 (PKH_101740)	40.6 (PBANKA_111490)	40.2 (PYYM_1117000)	39.6 (PCHAS_111450)
VPS15 (PF3D7_0823000)	39.3 (PVX_089215)	41.3 (PKH_050800)	46.0 (PBANKA_070760)	44.2 (PYYM_0707800)	44.2 (PCHAS_071690)
